# Magnetogenetics: remote activation of cellular functions triggered by magnetic switches

**DOI:** 10.1039/d1nr06303k

**Published:** 2021-11-15

**Authors:** Susel Del Sol-Fernández, Pablo Martínez-Vicente, Pilar Gomollón-Zueco, Christian Castro-Hinojosa, Lucía Gutiérrez, Raluca M. Fratila, María Moros

**Affiliations:** Instituto de Nanociencia y Materiales de Aragón (INMA), CSIC-Universidad de Zaragoza Zaragoza 50009 Spain lu@unizar.es rfratila@unizar.es m.moros@csic.es; Centro de Investigación Biomédica en Red de Bioingeniería, Biomateriales y Nanomedicina (CIBER-BBN) Spain; Departamento de Química Analítica, Universidad de Zaragoza Zaragoza 50009 Spain; Departamento de Química Orgánica, Universidad de Zaragoza C/Pedro Cerbuna 12 Zaragoza 50009 Spain

## Abstract

During the last decade, the possibility to remotely control intracellular pathways using physical tools has opened the way to novel and exciting applications, both in basic research and clinical applications. Indeed, the use of physical and non-invasive stimuli such as light, electricity or magnetic fields offers the possibility of manipulating biological processes with spatial and temporal resolution in a remote fashion. The use of magnetic fields is especially appealing for *in vivo* applications because they can penetrate deep into tissues, as opposed to light. In combination with magnetic actuators they are emerging as a new instrument to precisely manipulate biological functions. This approach, coined as magnetogenetics, provides an exclusive tool to study how cells transform mechanical stimuli into biochemical signalling and offers the possibility of activating intracellular pathways connected to temperature-sensitive proteins. In this review we provide a critical overview of the recent developments in the field of magnetogenetics. We discuss general topics regarding the three main components for magnetic field-based actuation: the magnetic fields, the magnetic actuators and the cellular targets. We first introduce the main approaches in which the magnetic field can be used to manipulate the magnetic actuators, together with the most commonly used magnetic field configurations and the physicochemical parameters that can critically influence the magnetic properties of the actuators. Thereafter, we discuss relevant examples of magneto-mechanical and magneto-thermal stimulation, used to control stem cell fate, to activate neuronal functions, or to stimulate apoptotic pathways, among others. Finally, although magnetogenetics has raised high expectations from the research community, to date there are still many obstacles to be overcome in order for it to become a real alternative to optogenetics for instance. We discuss some controversial aspects related to the insufficient elucidation of the mechanisms of action of some magnetogenetics constructs and approaches, providing our opinion on important challenges in the field and possible directions for the upcoming years.

## Abstract 2

Magnetogenetics: the use of magnetic fields along with magnetic actuators can be used to modulate biological functions in a non-invasive way, paving the way for the development of exciting tools useful in basic research and clinical applications.

## Graphical abstract

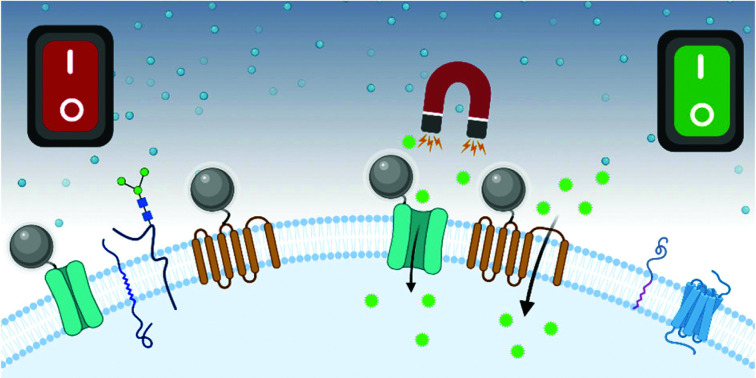

## 1. Introduction

Our cells utilize a set of receptors capable of perceiving physical cues from their environment, which are involved in physiological processes such as touch or nociception and in pathological processes like cardiomyopathies or cancer progression.^1^ During the last years, much effort has been devoted to the development of tools for remote manipulation of these cellular functions, using non-invasive stimuli such as light, electricity, ultrasound or magnetic fields. These technologies can contribute to shedding light on our understanding of biological processes, paving the way for the development of exciting tools useful in basic research and clinical applications.

Optogenetics for instance has provided great advances during the last decades for neuromodulation.^2^ This technique uses light to modulate cells that have been previously engineered to respond to those wavelengths, and is extremely useful because of its fast response and the availability of a large number of light-responsive receptors. However, when light has to reach deep structures, a fibre optic implant is routinely needed, as light in the ultraviolet and visible range does not penetrate well into the tissue.^3,4^ In order to overcome such problems, an emerging tool to control biological functions is based on the use of magnetic fields along with magnetic actuators, approach coined as magnetogenetics. The main advantage of this technique is that magnetic fields can penetrate deep tissues, which is especially relevant for *in vivo* applications. In addition, another advantage of this technique over optogenetics is the possibility to precisely modulate the external field, allowing a wide range of stresses and forces (fN to nN) to be applied without damaging the sample.

This approach has been extensively used to study mechanotransduction processes, that is, how cells respond to mechanical stimuli and convert them into biochemical signalling. In nature, mechanical stimulation of cells comprises phenomena such as compression, tension or fluid flow, each triggering different downstream cellular responses.^5^ In this context, many studies over the past decades have used magnetic microparticles and techniques such as magnetic tweezers or traction force microscopy to highlight how mechanical cues can impact biological processes.^6,7^ However, the size of magnetic microparticles results in multivalent binding, causing clustering of receptors and activation of intracellular signalling even in the absence of a magnetic field,^8^ preventing the required spatial control at the molecular level. Therefore, the use of smaller magnetic actuators such as ferritin or magnetic nanoparticles (MNPs), with sizes comparable to conventional proteins, permits a specific targeting of cell receptors. Such magnetic actuators, in combination with magnetic fields, are emerging as new instruments to precisely manipulate mechanical forces, providing an exclusive approach to the study of mechanotransduction. Although to a lesser extent, magnetic actuators have also been used for magnetothermal stimulation, activating intracellular pathways connected to temperature-sensitive proteins. Overall, this technology offers exciting opportunities for the manipulation of different functions *in vitro* and *in vivo* in a subtle way.^9^ Pioneering works during the last decade have used it to open ion channels,^10–13^ to regulate cell fate^14,15^ or even to manipulate individual receptors with exquisite control.^8^

The general idea behind magnetogenetics is that a magnetic actuator exposed to a magnetic field will induce a mechanical load or will generate heat, activating intracellular pathways. Therefore, there are three main components for the remote activation of cellular functions based on magnetic materials: (i) the magnetic field (that exerts a specific force or delivers energy to the actuator), (ii) the magnetic actuator (*e.g.*, MNPs (single core or clusters) or ferritin) and (iii) the target being activated at the cellular level. In particular, the actual mechanism involved in the magnetic activation of a biological receptors will depend on many parameters such as: (i) the type of magnetic fields being applied, either a static or a rotating direct current (DC) field, a DC gradient (static, pulsed or with some kind of movement) or an alternating current (AC) field, (ii) the magnetic properties of the magnetic actuator, and (iii) the intrinsic physical properties (thermal, mechanical, *etc*.) of the targeted cell receptors.

In this review we will discuss all these parameters, describing recent examples of magnetic actuators used for modulating cellular pathways. We will also discuss the recent discrepancies that can be found in the literature, highlighting that despite its great potential, there are still many issues that must be resolved before the high expectations initially raised by magnetogenetics can be reached.

## 2. Magnetic actuators and their manipulation with magnetic fields

### 2.1. Magnetic fields

It has to be noted that a MNP, with a given magnetic moment per particle (*μ*_NP_), when exposed to an external magnetic field (*H*) will be affected by the direction and amplitude of such field (see Box 1). The interplay between the magnetic moment and the field is the origin of the different possible mechanisms of magnetic manipulation.

Box 1 **Glossary of magnetism**

Ambiguities and confusion may occur in the description of “magnetic fields” and their corresponding units. Three main magnetic vectors are used when talking about “magnetic fields”: H, M and B. In the SI system, these three vectors are related as:

*B* = *μ*_0_ (*H* + *M*), where *μ*_0_ is the permeability of free space. *B* results from the sum of the magnetic field (*H*) and the magnetisation (*M*) of the medium. *B* is called magnetic flux or magnetic induction and its units in the SI are Tesla (*T*). *H* and *M* units are A m^−1^. This is the reason why when describing magnetic fields, some authors provide data in different units.

**•Magnetic field gradient**: Describes a situation where the magnetic flux lines are not parallel and converge or diverge within a region of space.

**•Magnetic anisotropy:** The dependence of magnetic properties on a preferred direction of a material is called magnetic anisotropy and depends on different parameters such as the particle shape, surface and crystalline structure. The effective anisotropy constant (*K*_eff_) of a MNP is a measurement of the material anisotropy taking into account all these three parameters. The anisotropy is responsible for the preferred orientations of the magnetic moment in the space.

**•Easy magnetisation axis**: Is the spatial direction inside a crystal, along which a small applied magnetic field is sufficient to reach the saturation magnetisation.

**•Hard magnetisation axis**: Is the direction inside a crystal, along which a larger applied magnetic field (in comparison with the easy axis) is needed to reach the saturation magnetisation.

**•Saturation magnetisation (*M***_**s**_ **)**: Is the maximum magnetic moment per unit volume for a magnetic material, reached when all magnetic moments are oriented parallel to the field.

**•Magnetic moment (*μ*)**: Is a vector that describes a dipole's ability to align itself with a given external magnetic field.

**•Superparamagnetism**: Is a magnetic behaviour of single-domain nanoparticles, originated from the fast-flipping process of the total magnetic moment due to thermal energy. In the absence of a magnetic field, the particles magnetic moments are randomly oriented, resulting in a negligible net magnetization, while in the presence of a magnetic field they will tend to align in the field direction creating a significant magnetization of the whole set of particles.

**•Ferrimagnetism**: Is a magnetic behaviour of materials in which the magnetic moments of unequal magnitude on different sublattices are arranged in an antiparallel way. Ferrimagnets have nonzero magnetisation in the absence of an applied field because their adjacent dipole moments do not cancel.

**•Dipolar interaction**: Is a type of long-range magnetic interaction occurring between two magnetic moments.

**Fig. 1:**
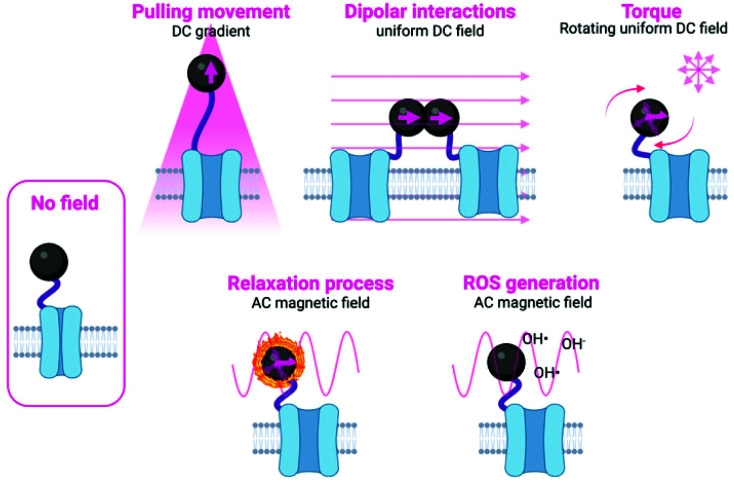
Schematic representation of the different mechanisms used to manipulate magnetic actuators, classified indicating the type of magnetic field (either DC or AC) and the type of activation being used. Figure created with *BioRender.com*.

**Fig. 2:**
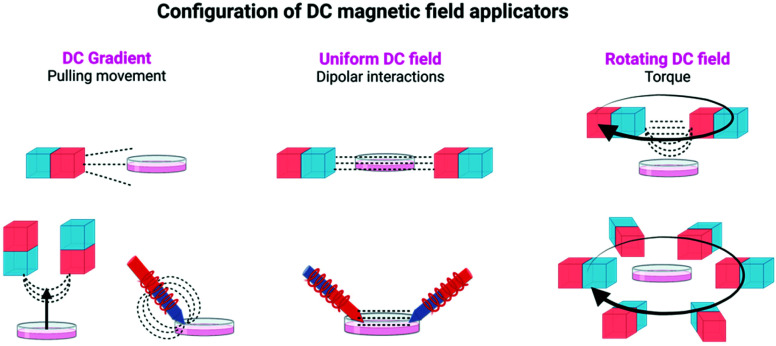
Different configurations of DC magnetic field applicators commonly used in magnetogenetic experiments, using either permanent magnets or electromagnets. Figure created with *BioRender.com*.

In general, both DC and AC fields have been employed for the manipulation of magnetic actuators. The main difference between them is that, for a given point in space kept at the same distance from the source generating the field, the DC field maintains its direction and magnitude over time, while an AC field periodically reverses its direction and changes its magnitude with time. Within the use of DC fields, several approaches have also been described (see Fig. 1, 2, and Table 1).

**Table 1:** Comparison table of magnetomechanical stimulation and receptor clustering reports

	Mechanism	Target	MNP size	Magnet configuration	Force per NP	Magnetic field	Ref.
1	Magnetomechanical (pulling movement and/or torque)	TREK1	300 nm (Micromod)	NdFeB array (bioreactor)	Not disclosed	∼75 mT–1.4 mT	97
						1 Hz	
2		Frizzled	250 nm (Micromod)	NdFeB array (bioreactor)	Not disclosed	∼25 mT (max)	34
						1 Hz	
3		Frizzled	250 nm (Micromod)	NdFeB array (bioreactor)	Not disclosed	25–120 mT	15
						0.9–1 Hz	
4		TREK1, Integrins	300 nm (Micromod)	NdFeB array (bioreactor)	4 pN	25 mT (max)	96
						1 Hz	
5		PDGFRα, Integrins	250 nm (Micromod)	NdFeB array (bioreactor)	10–30 pN	60–120 mT	137
						1 Hz	
6		TREK1, Integrins	250 nm (Micromod)	NdFeB array (bioreactor)	1–100 pN	Not disclosed	82
						1 Hz	
7		Frizzled	250 nm (Micromod)	NdFeB array (bioreactor)	pN	>25 mT	35
8		TRPV4	100 nm (Chemicell)	NdFeB (static)	∼25–33 pN	∼110 mT	55
9		Glycoproteins	45 nm (cubic Zn_0.4_Fe_2.6_O_4_)	Electromagnetic needle	0.1 pN	1000 T m^−1^ (10 μm from the tip)	47
10		Piezo1	∼31.5 nm (iron oxide MNPs)	NdFeB (static)	Not disclosed	70–80 mT	36
11		TRPV4	226 nm (magnetic vortex nanodiscs)	Electromagnet	140 pN	≤28 mT	52
						5 Hz	
12		Piezo1	500 nm (25 nm octahedral MNPs assembled on polystyrene beads)	NdFeB array (rotating)	2 pN	>20 mT (uniform, ∇*B* < 10 T m^−1^)	53
13		Exogenous magnetoreceptor, iron–sulphur cluster assembly protein 1	Not disclosed	Electromagnet	Not disclosed	1–2.5 mT	109
14		TRPV4 fused to ferritin	Not disclosed	NdFeB array	Not disclosed	50 mT-250 mT	13
15	Receptor clustering (dipolar interaction)	IgE receptors (FcεRI)	30 nm-5 nm iron core (Nanocs)	Electromagnetic needle	1 × 10^−5^ pN	Not disclosed	11
16		Tie2 receptors	15 nm (Zn^2+^-doped ferrite MNPs)	2 permanent NdFeB magnets (static)	1 × 10^−5^ pN	∼150 mT	48
17		Ovarian tumor necrosis factor receptor (OTR)	15 nm (Zn_0.4_Fe_2.6_O_4_ MNPs)	2 permanent NdFeB magnets, 1 cm gap (static)	(a) 9.2 × 10^−7^ pN (individual MNP) (b) 0.034 pN (interparticle force between 2 MNPs)	500 mT	12
18		Death receptor 4 (DR4)	15 nm (Zn_0.4_Fe_2.6_O_4_ MNPs)	2 electromagnetic coils	Not disclosed	500 mT	113
19		EGFR	12–15 nm (iron oxide MNPs)	4 NdFeB magnets in a quadrupole configuration (static)	∼0.25 pN (force between 2 MNPs)	Not disclosed	114
20		Major histocompatibility complex (MHC) and CD28	50–100 nm (iron-dextran NPs)	2 NdFeB magnets (static)	Not disclosed	200 mT	115
21		MHC and CD28 or CD27	30–500 nm (iron-dextran NPs)	2 NdFeB magnets (static)	Not disclosed	Not disclosed	116

In static homogeneous DC fields, the direction and the magnitude of the field are the same in different points in space and are kept constant over time. In rotating DC fields, the direction of the magnetic field in a given point in space changes with time, while its magnitude is maintained. Finally, magnetic field gradients, in which the direction and/or the magnitude of the field is different in two points in space, are also commonly used in magnetogenetics experiments. To generate these magnetic fields, permanent magnets or electromagnets with a multitude of spatial and electronic configurations can be used (see Table 1, Fig. 1 and 2, and ref. 16–18 for more details).

The main approaches in which the magnetic field can be used to manipulate the magnetic actuators can be classified into two subgroups: mechanical and thermal activation. In the subgroup of mechanical activation, the main possibilities proposed until now are pulling movement, dipolar interactions and torque (Fig. 1 and Table 2).^9^ These hypotheses have been tested either experimentally or theoretically, although often the exact mechanism taking place is unknown or more than one mechanism occurs at the same time. In the subgroup of the thermal activation, a heating process occurs as a result of relaxation mechanisms. In addition to these two different activation possibilities, some other researchers have proposed other alternatives, such as the generation of Reactive Oxygen Species (ROS).^19^ Details on each specific approach are described below and schematically depicted in Fig. 1. Moreover, an overview of the main advantages and disadvantages of the principal stimulation mechanisms is provided in Table 2.

**Table 2:** Overview of the main advantages and disadvantages of the principal stimulation mechanisms

Stimulation	Advantages	Disadvantages
Pulling movement	Easy set-up when using permanent magnets	Very large gradients are needed
	The application of a broad range of parameters is possible when using electromagnets	Difficult to implement when the magnetic actuator is ferritin. Therefore, synthetic MNPs with large magnetic moments (which is proportional to the MNPs volume) are better suited for this type of stimulation
	Many examples can be found in literature, for a great diversity of cellular targets	Refrigeration may be needed when using electromagnets (due to overheating)
	The stimulation can be intra- and extracellular	Working distance is usually short (generally in the micron range)
	Demonstrated *in vitro* and *in vivo*	
Dipolar interactions	Collective magnetic behaviour is favoured, enhancing the net generated force	Uniform fields in large areas may be difficult to achieve
	Demonstrated *in vitro* and *in vivo*	It is difficult to precisely control the distances among particles to allow their clustering
		Receptors need to be stimulated by clustering (fewer examples in the literature)
Torque	Long-distance stimulation is possible (maximal reported working range up to 70 cm)	This is the least studied mechanism in magnetogenetics
	Low magnetic field strength is needed to generate a large torque	The field applicator device is complex and for some configurations there are no commercially available options, which hampers the comparison of results between different reports
	Previous knowledge regarding this mechanism is available for other applications (e.g., single molecule manipulation)	
	Demonstrated *in vitro* and *in vivo*	
Relaxation process	Heating of magnetic nanoparticles under AC fields has been widely studied in the frame of magnetic hyperthermia	There are less receptors prone to thermal activation (in comparison with mechanical activation)
	AC field generators are commercially available	Response times can be somewhat slow for neuromodulation when compared to mechanical stimulation

#### 2.1.1. Pulling movement (magnetic field gradient)

When placed in a magnetic field gradient, magnetic actuators will move towards the source of the field.^16^ If the particles are linked to a cell receptor, their movement will generate a pulling force.

The strength of such force will depend on the magnetic moment of the particle and the magnetic field gradient. In this approach, the rate and magnitude of the applied force are also important, as the frequency of pulling movements has been related to different responses from cells.^18^ To achieve this type of movement by the particles, generally strong magnets (composed of Nd, Fe and B and often called neodymium magnets) are used, either in arrays or as single magnets, although electromagnetic needles have also been described. NdFeB magnets included in vertical oscillating magnetic force bioreactors have been extensively used for magnetogenetics experiments (see Table 1 and Fig. 2).

#### 2.1.2. Dipolar interactions (static DC magnetic field)

If particles are placed under a constant DC field, their magnetic moments will tend to align in the direction of such field. If particles are located relatively close to each other, they may experience a dipole–dipole attraction force, depending again on their magnetic moments, the distance between particles, the global temperature and the field applied. Dipolar interactions decrease very fast with the inter-particle distance (*r*). Therefore, for this mechanism to be feasible, the particles need to be very close together, in the nanometer range.^20^ In this case, the movement will be of particles approaching each other. This can be explored to activate the controlled clustering of membrane receptors where magnetic actuators are attached (see section 6).^12^ Static DC fields have been mainly generated using permanent magnets although in some cases electromagnets have also been used (see Table 1 and Fig. 2).

#### 2.1.3. Torque (rotating DC field)

If a particle is placed within a constant DC field, but the direction of such field rotates over time, the particle (or its magnetic moment) may rotate in order to align with the field direction. This rotation movement can generate a torque able to activate the selected cell targets.^21^ In this kind of approach, working with single particles or aggregates, in which the particles display randomly oriented axes, may change dramatically the final effect.^22^ Also, the relationship between the direction of the applied field and the easy axis of the magnetic actuator will play an important role on the final torque generated.^22^ To generate the field required for the torque, the mechanical rotation of NdFeB magnets is routinely used (see Table 1 and Fig. 2).

#### 2.1.4. Relaxation process (AC magnetic field)

In this approach, the rotation of the magnetic moments in order to follow the continuous change of direction of the AC field results in a release of energy in the form of heat, with the subsequent increase of the local temperature.^23^ This is the same mechanism as the one used in magnetic hyperthermia for cancer treatment.^24^ For this approach a coil able to generate the AC fields is needed (see Table 3).

**Table 3:** Comparison table of magnetothermal stimulation reports

		Target	MNP inorganic core diameter/composition	AC magnetic field	Ref.
1	Magnetothermal	TRPV1	6 nm/MnFe_2_O_4_	0.67 kA m^−1^; 40 MHz	46
2		TRPV1	22 nm/Fe_3_O_4_	15 kA m^−1^; 500 kHz	111
3		TRPV1	25 nm (Ocean nanotech)/iron oxide	15 kA m^−1^; 500 kHz	37
4		TRPV1	10 nm/CoFe_2_O_4_ core - MnFe_2_O_4_ shell	22.4 kA m^−1^; 412.5 kHz (*in vitro*); 7.5 kA m^−1^; 570 kHz (*in vivo*)	58
5		Ano1/TMEM16A	∼13 nm/MnFe_2_O_4_ core – CoFe_2_O_4_ shell	28.9 kA m^−1^; 412.5 kHz	50
6		TRPV1	20 nm (Ocean nanotech)/iron oxide	4 kA m^−1^, 465 kHz	39
7		TRPV1	∼16 nm/Fe_3_O_4_, ∼18 nm/Co_0.24_Fe_2.76_O_4_	10 kA m^−1^; 522 kHz (for Fe_3_O_4_); 70 kA m^−1^; 50 kHz (for Co_0.24_Fe_2.76_O_4_)	49
8		TRPV1	GFP-ferritin	24, 21 or 18 kA m^−1^; 465 kHz	104
9		TRPV1	Ferritin nanoparticles	25 or 23 kA m^−1^; 465 kHz	110

#### 2.1.5. Generation of reactive oxygen species (AC magnetic field)

It has been observed that iron oxide nanoparticles generate ROS through the Fenton reaction^25^ triggering cation channel activation.^19^ This phenomenon can even be increased by the application of an AC magnetic field.

Some of these approaches remain controversial, especially when the magnetic actuator is ferritin, whose magnetic moment is much weaker than that of iron oxide MNPs. In fact, there are still many not well-known parameters that hinder a complete knowledge of the processes occurring during magnetogenetics experiments. Among these parameters are the magnetic properties of ferritin iron cores (when used as magnetic actuator) and the physical properties (thermal, mechanical, and diamagnetic) of ion channels and cell membranes coupled to the iron-loaded ferritins^26^ (see section 7 for a more detailed discussion).

### 2.2. Magnetic actuators

Small magnetic actuators offer undeniable advantages for manipulating cellular pathways, as they can be stimulated in a remote and spatiotemporal fashion, and at the same time they show spatial control at the molecular level.^27^ One limitation, however, of using magnetic actuators instead of microparticles is that the exerted forces are in the range of fN or pN.^4,9^ While small and isolated MNPs exert weak force ranges (fN or hundreds of fN), clusters of MNPs can arrive to strong force ranges (sub-nN).^9^ On the other hand, cellular events such as spatial clustering, conformational changes or mechanical activation of receptors require forces in the range of sub-pN to hundreds of pN (see section 3).^9^ Therefore, the magnetic properties of the actuator and the design of the magnetic field applicator are crucial for the success of magnetogenetics applications.

Two main possibilities on the use of magnetic actuators for magnetogenetics applications are described in the literature. The first one is the use of MNPs, mainly composed of iron oxides or doped ferrites, given the low toxicity of iron in comparison with other “magnetic” elements such as Co^2+^ or Ni^2+^ and their tuneable magnetic properties. The second approach is the use of ferritin-based magnetic actuators, in which the biomineralized iron oxide or iron oxyhydroxide nanoparticles located inside the protein shell are the ones with interesting magnetic properties. In all cases, the magnetic properties of the iron-containing core are the ones that are relevant for the magnetogenetics application, limiting the forces that can be exerted by the external magnetic field.

**Fig. 3:**
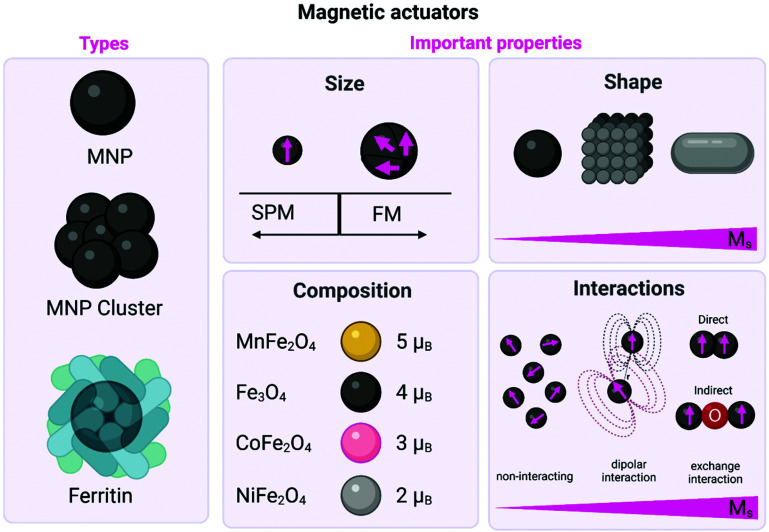
Schematic representation of the different types of magnetic actuators including both MNPs (either single core or clusters) and ferritin. The main physicochemical properties that affect their magnetic behaviour are also described including: (i) the influence of the size on the domain structure and the onset of superparamagnetism (SPM) and/or ferrimagnetism (FM) state; (ii) the impact of the shape on the *M*_s_ values; (iii) the impact of the composition on the magnetic moment per unit cell in the ferrite structure; and (iv) the impact of the interactions on the *M*_s_ values. Figure created with *BioRender.com*.

Some physicochemical parameters that must be taken into account and can critically influence the magnetic properties of the actuators are their size, composition, shape and magnetic interactions of the actuators (Fig. 3). These parameters have a fundamental impact on their saturation magnetisation values (*M*_s_), their effective anisotropy (*K*_eff_), the magnetic moment per particle (*μ*_NP_), or the magnetic moment per iron atom (*μ*_Fe_) (see Box 1).

#### 2.2.1. Iron oxide and doped ferrite nanoparticles

##### 2.2.1.1. Size

MNPs lack the multidomain structures present in bulk magnetic materials, giving rise to single magnetic domain structures and the onset of superparamagnetism (SPM), (Fig. 3, Box 1).^28^ For example, the particle size required to achieve SPM in Fe_3_O_4_ MNPs is widely estimated to be below 20 nm in diameter.^29–31^ Large MNPs present a multidomain structure, where each domain exhibits uniform magnetisation and is separated from its neighbours by domain walls. As the particle size is decreased (with the corresponding increase in the surface-to-volume ratio), there is a point where domain wall creation is no longer energetically favourable, preventing the existence of the multidomain structure. Hence, the nanoparticle becomes single domain, with all atomic spins aligned in the same direction. The single domain limit appears at the critical particle diameter, which is characteristic for each material, and depends on their anisotropy and exchange constants, as well as their *M*_s_ value. In this sense, the critical size for forming a multidomain structure has been theoretically estimated to be 76 nm for cubic and 128 nm for spherical Fe_3_O_4_ MNPs.^29,32^ Working with superparamagnetic nanoparticles is fundamental for biomedical applications, as these particles will not present a net magnetisation in the absence of an external magnetic field, preventing agglomeration processes that could occur with bigger particles.

An extensive overview of several magnetic actuators and their correlation with the mechanical force that they can exert under different magnetic fields is presented in Table 1. For example, it was shown that classical iron oxide microparticles which are commercially available (2.8 μm; Dynabead) coated with wheat germ agglutinin (WGA) can generate 30 pN per microparticle under a rotating DC field of 200 mT.^33^ Other big magnetic actuators, such as 250 nm magnetite MNPs were capable of activating intracellular pathways in a neuronal cell line^34^ and in mesenchymal cells^35^ by generating mechanical forces of about 10–12 pN. Moreover, a positive correlation between size and exerted mechanical force was reported for a set of magnetite particles ranging from 250 nm to 2.7 μm in diameter, which could generate mechanical forces between 0.2 to 38.9 pN per particle depending on their size.^10^

However, as mentioned before, microparticles suffer from disadvantages associated with their large size, such as low target labelling density, multivalent binding with target receptors, high non-specific binding and also, that their mode of force stimulation is mainly limited to pulling movement or/and torque.^8,9^ In this sense, small MNPs present several advantages such as the multiple modes of force stimulation (see section 2.1), the monovalent binding with target receptors and the high labelling density due to their high surface area.^8^ For instance, small superparamagnetic Fe_3_O_4_ MNPs with 9.1 ± 2.8 nm average diameter and a *M*_s_ of 46.1 Am^2^ kg^−1^ of MNPs mixed in a PLGA matrix were able to stimulate the mechanosensitive protein Piezo1 and accelerate osteogenesis under exposure to a static DC field (70–80 mT).^36^ Sometimes, large differences can be found in the receptor activation using MNPs of similar size. For instance, MNPs between 10 and 30 nm have been studied for the excitation of neurons following magneto-thermal drug release and subsequent activation of the thermosensitive transient receptor potential vanilloid family member 1 (TRPV1) channel (see also section 5).^37^ Although both the 20 and 25 nm MNPs had similar specific loss powers, the 25 nm MNPs exhibited a twice-higher intrinsic particle loss power, due to their higher magnetic diameter and thus, enhanced magnetic susceptibility compared with their 20 nm counterparts. Therefore, the 25 nm MNPs were considered to be the most suitable actuators for pharmacological excitation under exposure to an alternating magnetic field (AMF) of 15 kA m^−1^ and 500 kHz. These AMF parameters fulfill the therapeutic criteria for AMF application, according to which the product amplitude-frequency should be below 5 × 10^9^ Am^−1^ s^−1^.^38^

In a similar approach, iron oxide MNPs between 10 and 50 nm were tested to induce TRPV1 thermal activation under an AMF (465 kHz and 5 mT). Under those conditions, the highest temperatures were achieved with the 20- and 25 nm MNPs.^39^

It is worth mentioning that there are several reports in the literature detailing the impact of MNP size on the magnetic properties of particles for biomedical applications, such as magnetic hyperthermia and magnetic resonance imaging.^40–42^ However, a systematic investigation of the relationship between magnetic domain structures and the exerted mechanical forces is still lacking.

##### 2.2.1.2. Composition

Spinel metal ferrites (MFe_2_O_4_ where M = Fe^2+^, Mn^2+^, Zn^2+^, Co^2+^, *etc*.) can be considered as close-packed cubic arrays of oxygen ions with tetrahedral (T_d_) and octahedral (O_h_) sites occupied by the metal cations. The unit cell contains 32 oxygen ions, 16 Fe^3+^ ions and 8 Fe^2+^ ions. Fe^2+^ ions occupy one quarter of the O_h_ sites, while other quarter is occupied by 8 Fe^3+^ ions. The rest of the Fe^3+^ ions fill in one eighth of the T_d_ sites. Under an external magnetic field, spins in O_h_ sites align in parallel with the direction of the field, while those in T_d_ sites align antiparallel. Since spins in both lattices are generally uncompensated, the resulting net magnetic moment causes the material to display ferrimagnetic behaviour.^43^ Replacing Fe^2+^ ions with a magnetic moment of 4 *μ*_B_ by Mn^2+^ or Co^2+^ ions, the net magnetic moment of the MFe_2_O_4_ unit cell can be tuned from 4 *μ*_B_ to 5 or 3 *μ*_B_, respectively (Fig. 3).^27,44^ Meanwhile, the introduction of low amounts of Zn^2+^ ions reduces the unbalance between antiferromagnetic coupling of Fe^3+^ ions in the T_d_ and O_h_ sites, and thus leads to incremental changes in the *M*_s_.^45^

This strategy of substituting metal dopants in ferrite nanoparticles to achieve tunable magnetocrystalline anisotropy has been used to exert high mechanical forces (in the pN range), resulting in the remote control of ion channels^46,47^ and the activation of mechanoreceptors.^8,12,48^ Among others, zinc–iron oxide nanoparticles are so far the most explored nanoparticles for mechanogenetic applications. In this sense, Cheon *et al.*^12^ designed spherical 15 nm zinc-doped iron oxide NPs (Zn_0.4_Fe_2.6_O_4_) with an exceptionally high *M*_s_ value (161 Am^2^kg_Fe_^−1^) coated with a targeting antibody for death receptor 4 (DR4) in colon cancer cells. Under exposure to a permanent magnetic field (0.20 T), the calculated interparticle attraction force was about 30 fN, enough for effective clustering of death receptors on the cell membrane (see section 6). Additionally, MNPs composed by the same Zn_0.4_Fe_2.6_O_4_ magnetic core, but coated with a silica layer and a gold shell (Zn_0.4_Fe_2.6_O_4_@SiO_2_@Au), with an average diameter of 50 ± 4 nm were employed for mechanical stimulation of Notch or VE-cadherin receptors.^8^ Even though the *M*_s_ value and the magnetic behaviour of the MNPs were not described in detail, mechanical forces of about 1 pN and 9 pN for weak and strong force modes, respectively, were reported.

**Fig. 4:**
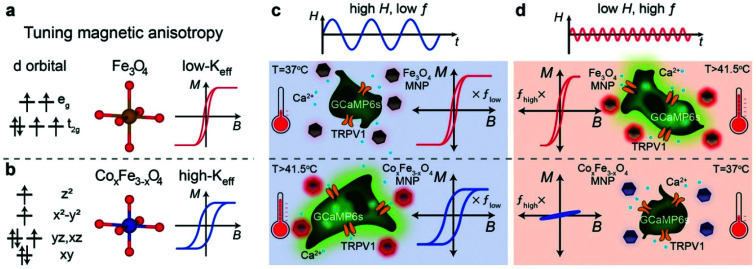
(a and b) Schematic illustration showing the concept of selective magnetothermal stimulation of cells using MNPs with different magnetic anisotropy. (c and d) Response of each type of MNP to different AMF conditions and subsequent effect on TRPV1 thermal activation. Reprinted with permission from J. Moon, *et al.*, *Adv. Funct. Mater*., 2020, **30**, 2000577. “Magnetothermal Multiplexing for Selective Remote Control of Cell Signaling”, copyright (2020) WILEY-VCH Verlag GmbH & Co. KGaA, Weinheim.

Other doped ferrites such as manganese ferrite (MnFe_2_O_4_) and cobalt ferrite (CoFe_2_O_4_) nanoparticles have been also used for magnetothermal stimulation of cation channels.^46,49,50^ Interestingly, Moon *et al.*^49^ showed that the combination of the magnetic properties of CoFe_2_O_4_ MNPs (high coercivity and low *K*_eff_) and Fe_3_O_4_ MNPs (low coercivity and high *K*_eff_) with specific AMF conditions (high amplitude and low frequency for CoFe_2_O_4_*versus* low amplitude and high frequency for Fe_3_O_4_ MNPs) can provide a selective trigger for TRPV1 opening, and thus, Ca^2+^ influx (Fig. 4).

##### 2.2.1.3. Shape

Shape anisotropy appears as a result of deviations from a symmetric shape, like a perfect sphere. This source of anisotropy dictates a preferred orientation of the *μ*_NP_ with respect to the major/minor axes of the particle. The modification of MNP shape may also lead to an increase in the *K*_eff_ as surface effects become more important. The cube has the lower energy surface facets of the family; in contrast, the surface of a spherical nanoparticle is constructed of different facets, which results in a larger surface spin disorder, hence higher surface anisotropy. Thus, faceted or cubic-shaped MNPs display higher *M*_s_ and heating efficiency values than their spherical counterparts.^51^ For instance, Gregurec *et al.*^52^ designed magnetic vortex nanodiscs (MNDs) with different sizes (98 to 226 nm) for magnetomechanical activation of transient receptor potential vanilloid family member 4 (TRPV4). Two sizes of MNDs were selected (98 nm and 226 nm) and coated with poly(maleic anhydride-*alt*-1-octadecene) to achieve stability in physiological fluids and facilitate their attachment to cell membranes. Under exposure to slowly varying (≤5 Hz) magnetic fields (≤28 mT), the vortex disks were magnetized in a direction compatible with their easy axes (on the plane of the disk, see Box 1), which generated the rotation of the MNDs and the concomitant mechanical torque. These MNDs exhibited high *M*_s_ value (115–120 Am^2^ kg_Fe_^−1^) and exerted torque forces in the order of 140 pN for the biggest nanodisk and assuming completely in plane magnetisation (see section 4). 25 nm well-faceted octahedral MNPs assembled on a 500 nm spherical polystyrene support have also been explored to activate Piezo1 ion channels in *in vitro* and *in vivo* models. The MNPs displayed high *M*_s_ values (167 Am^2^ kg_Fe_^−1^) and generated 2–10 pN at 20–50 mT.^53^ Lastly, the cube-like morphology has been also explored as an effective actuator for mechano-gating of ion channels on the cell membrane.^36,47,54^ However, digging in the literature, only few articles deal with the tuning of the shape as a source of anisotropy to improve the magnetic actuator behaviour in mechanogenetic applications, while the effect of shape anisotropy for thermal activation has not been described. Moreover, the impact of other morphologies such as rods or flowers is still an unexplored field in magnetogenetics.

##### 2.2.1.4. Magnetic interactions

Collective assemblies of nanoparticles, summing up their individual magnetic moments, represent another approach to boost the total magnetic moments. For MNPs, there are two types of magnetic interactions: dipolar interactions (generally between the magnetic moments of different particles) and exchange interactions (between spins in close contact) which can be direct interactions between two magnetic ions or indirect interactions mediated *via* a non-magnetic ion (see Fig. 3). For example, Tay *et al.* reported that the mechanical force generated by an individual starch coated MNP was between 25–33 pN.^55^ However, considering the formation of clusters of about 1.6 μm, formed by the same starch coated MNPs, inside the cell body of a neuron, the mechanical force scaled up to 52 pN. The authors found a positive correlation between the increased magnetic force generated by clusters and the influx of Ca^2+^, probing that the mechano-sensitive channels were opening. In another interesting report, ferrimagnetic nanoparticles with different sizes (between 110 and 280 nm) and coatings (starch, chitosan or glucuronic acid), were tested under several experimental conditions, such as, different field strengths, orientations, amounts and sizes of MNP clusters (between 1 to 5 clusters by neurons, from <0.5 μm^2^ to >2.0 μm^2^). It was then possible to produce a large combination of forces: from 4.3 pN to ∼1 nN in cortical neurons. By tuning the interacting ferrimagnetic nanoparticles-mediated forces and the other conditions mentioned above, the authors found optimal force ranges for intracellular redistribution of the microtubule-associated protein tau (4.5–70 pN), for tau repositioning (larger clusters, 190–270 pN) and for the initiation of cell displacement at forces above 300 pN.^56^

Regarding exchange interactions, versatile combinations of core–shell components can provide an easy adjustment of *K*_eff_, as well as of *M*_s_.^57^ In this sense, Munshi and coworkers designed exchange-coupled core–shell nanoparticles composed by a CoFe_2_O_4_ core and a MnFe_2_O_4_ shell that effectively opened TRPV1 channels in freely moving mice with as low as 500 ng of MNPs (for more details, see section 5).^58^ Varying the composition of the core–shell architecture, with MnFe_2_O_4_ as core and CoFe_2_O_4_ as shell, the same authors reported magnetothermal activation of thermosensitive chloride channels.^50^

#### 2.2.2. Ferritin-based magnetic actuators

The use of magnetic fields interacting with ferritin-based magnetic actuators also provides a rapid and non-invasive way for regulating cell activity.^3^ Ferritin is a protein that stores iron in the body and releases it in a controlled fashion. It is composed by 24 peptide subunits assembled into a hollow spherical shell where iron can be stored in a biomineral form. The iron content of the ferritin core is typically described as a ferrihydrite nanoparticle of up to 8 nm. However, the exact organization of the crystalline structure of the iron atoms in ferrihydrite and, therefore, the magnetic properties of ferritin remain under discussion.^59–61^ Moreover, the iron-containing core inside ferritin may be a single particle or a multicore structure,^62^ making even more complicated to elucidate the magnetic properties of this iron-containing protein. The magnetic characterization of some ferritins has previously reported magnetic moments per iron ion below 5 *μ*_B_, in agreement with some degree of antiferromagnetic interactions.^63^ However, studies on the aging of ferrihydrites, the crystalline structure generally associated to the ferritin core, have reported magnetic moments per iron ion up to 120 *μ*_B_, opening the possibility of some ferrimagnetic ordering and, as a consequence, a stronger magnetic behaviour.^59^

Different systems based on iron-containing particles located inside apoferritin structures have been generated for magnetogenetic applications. For example, Liße *et al.*^64^ reported a magnetoferritin platform composed by a monomeric enhanced green fluorescent protein fused to ferritin and a magnetite core of 7 nm synthesized inside. The system exhibited a high *M*_s_ value of about 87 Am^2^ kg^−1^ of MNPs (higher than ferrihydrite-loaded ferritin purified from horse spleen, *M*_s_ < 20 Am^2^ kg^−1^) and produced a mechanical force in the fN. Additionally, genetically modified ferritin monomers fused to proteins that can heterodimerize between them can yield micrometric clusters of ferritin.^65^ In this case, the magnetic properties were conferred due to the biomineralization of iron oxide MNPs into ferritin cavities (5 ± 1 nm). As a consequence of the collective magnetic behaviour of ferritin clusters, a high mechanical force of about 10 pN (cluster formed of about 104 ferritins) was reported.

Magnetic gating of ion channels has also been shown by inserting a ferritin-binding motif into transient receptor potential (TRP) channels under exposure to a weak (5 mT) AMF (465 kHz).^39^ However, some reports evidence no temperature increase on the protein surface or in the surrounding fluid due to the interaction of horse spleen ferritin (2 nm thick protein shell-coated 8 nm ferrihydrite core) with magnetic fields.^58,66,67^

In summary, regarding the use of ferritin as a magnetic actuator in magnetogenetics, additional experimental and theoretical studies are necessary to uncover the biophysical properties of this interesting protein, paying special attention to the size, shape and exact composition of its iron-containing core, which are key parameters that will determine its magnetic properties. This knowledge is fundamental to understanding the role and limitations of the use of this magnetic actuator in magnetogenetic applications.

## 3. Cellular targets for magnetogenetics

**Fig. 5:**
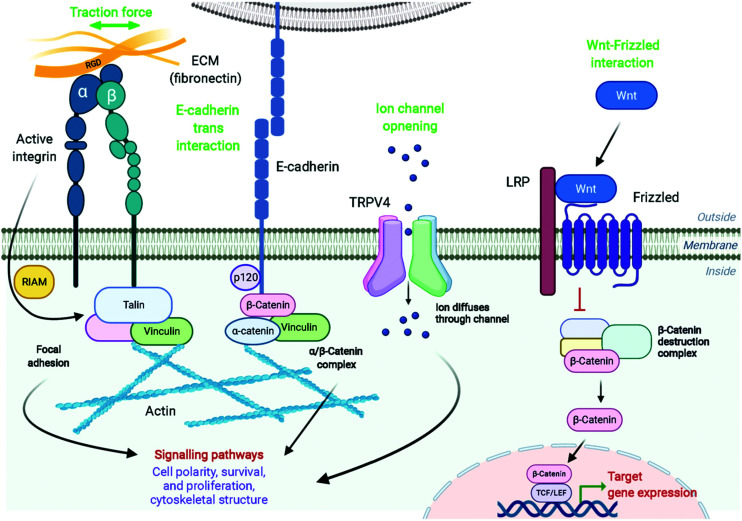
Examples of diverse membrane receptor signalling pathways that can be manipulated using magnetic actuators, including integrins, E-cadherin, TRPV4 and Frizzled. The physiological mechanisms of activation of these receptors are shown in green. See text for further details. Figure created with *BioRender.com*.

The magnetic actuators used for controlling cellular functions generally act in two main ways, *via* mechanical activation or *via* thermal stimulation.^3,4^ This remote manipulation can promote changes at the cellular membrane or inside the cell, modulating different cell functions like migration, contraction, secretion, proliferation or differentiation among others. All these processes can occur through many different mechanisms, such as conformational changes in proteins in contact with the target receptor, transmission of force to cytoplasmic mechanosensors, remodelling of the cytoskeleton conformation and/or participation of transcription factors that go into the nucleus and regulate the expression of target genes (Fig. 5).^4,68,69^

Cellular receptors/targets commonly stimulated by magnetic actuators can be grouped according to their biological functions and/or the signalling pathways that they modulate. Prominent examples of targets are: (i) cellular receptors such as Notch1 or Frizzled which activate important signalling pathways (Notch and Wnt, respectively), inducing the expression of downstream target genes, (ii) adhesion molecules like integrins or cadherins, or (iii) ion channels such as Piezo1, TWIK-related potassium (TREK1), TRPV1 or TRPV4, among others. Further, MNPs can also be internalized and guided with a magnetic field in order to activate other types of proteins such as the Rho GTPase family.^70^ The range of magnetic forces that are necessary for activating mechanotransduction processes depends on different parameters, such as the type of target, the number of magnetic actuators, their conformation (single *vs.* aggregated) or the mode of force stimulation among others. For example, the physiological stimulation of Notch1 is promoted by ligand-binding forces of 1 to 20 pN,^71^ whereas the interaction between integrins and fibronectin has a strength around 50 pN (single molecule measurements).^72^ Some of the most common cellular targets are explained below.

There is a complex interplay between adhesion receptors and the cytoskeleton, suggesting physical connection between membrane sensors and the internal cell scaffold. Integrins conform an important family of heterodimer transmembrane glycoproteins that transduce force or mechanical stimuli into biochemical signals. Integrin extracellular domains bind to ligands in the matrix, like fibronectin or collagen, and support cell adhesion,^73,74^ whereas their intracellular regions associate with the cytoskeleton, regulating among others cell survival, differentiation, migration, and mechanotransduction pathways.^75,76^ The mechano-stimulation of integrins promote, though activation of Rap1-GTP interacting adaptor molecule (RIAM1), the recruitment of essential adaptor proteins like talin and vinculin, and the scaffolding of F-actin microfilaments (Fig. 5).^77,78^ This cytoskeleton dynamics activates cell remodelling and downstream signalling, being the small Rho GTPases vital mediators of this pathway.^79^ Several studies of proliferation and osteogenic differentiation of stem cells have employed integrins as targets for magnetic actuators functionalized with RGD (see section 4).^14,80–82^ The RGD peptide is present within extracellular matrix proteins such as fibronectin, fibrinogen or osteopontin among others, and represents their principal binding domain to integrins.^74^ Therefore, the RGD peptide is routinely used to functionalize magnetic actuators in order to target integrins. Although not so commonly, some Rho GTPases have been intracellularly activated using magnetic actuators (see section 6).

In addition to integrins, other cell–cell adhesion receptors, like E- and VE-cadherins, can be targeted with magnetic actuators. Cadherins are transmembrane proteins with a Ca^2+^-dependent activity, that interact homophilically with other cadherins through their extracellular region. Cadherins are widely expressed in different cell types, playing an essential role in processes such as tissue and organ morphogenesis, cell–cell adhesion and mechanotransduction.^83,84^ The principal cytoplasmic binding partners of cadherins are p120, β- and α-catenins. p120 selectively regulates cadherin endocytosis and stability, and Rho GTPases family activity. Furthermore, cadherins are linked to the cytoskeleton *via* β-catenin-α-catenin-actin complex, with an integrin-cadherin adhesive crosstalk taking place.^85^ Studies in which MNPs have been used to target cadherins have revealed important information; for instance, it has been described that the magnetic force application promotes the recruitment of F-actin and vinculin to the cadherin-MNP clusters, stabilizing the cadherin-cytoskeletal complexes (Fig. 5).^8,86^ In addition to VE- and E-cadherins, the same group investigated the cell surface activation of another classical mechanoreceptor, Notch1, whose activation promotes downstream signalling.^8^ Notch receptors are single-pass transmembrane heterodimers composed of an extracellular part that binds to Jagged-Serrate or Delta-like receptors, a transmembrane region, and an intracellular portion. Upon interaction with its ligands, the intracellular domain of Notch1 is cleaved and converted in a nuclear transcriptional co-activator, which mediates signalling pathways involved in numerous physiological, developmental, and pathological processes.^87^ Interestingly, Seo *et al.* described that Notch1 can be also activated without the need of ligands, just by using MNPs and a strong force mode.^8^ While at 1 pN pulling force the MNPs bound to the targeted location and did not dissociate, at 9 pN of critical force, the MNPs were detached with the Notch extracellular domain, promoting the activation of the signalling pathway.

Other important signalling pathway that can be manipulated with magnetic actuators is Wnt, which is involved in the regulation of diverse biological processes, such as embryogenic development, cell proliferation and differentiation, tissue formation and regulation of stem cell fate.^88,89^ Wnt glycosylated protein interacts with the receptor complex composed of Frizzled/Low density lipoprotein receptor-related protein (LRP) located on the cell membrane, causing the initiation of a signalling cascade that leads to the accumulation of active β-catenin in the cytoplasm and finally in the nucleus. Nuclear β-catenin acts as a transcriptional regulator, interacting with the T-cell factor/lymphoid enhancer factor (TCF/LEF) family of transcription factors (Fig. 5).^90^ Wnt signalling plays an important role in the osteoblast differentiation of human mesenchymal stem cells (hMSCs), which has potential in bone and cartilage tissue engineering.^91^ Also, Wnt signalling is a key pathway that regulates neuronal differentiation during development and dopaminergic progenitor cell proliferation, representing a possible therapeutic target for neurodegenerative diseases like Parkinson's.^34^ Therefore, the effects of the Wnt-Frizzled receptor mechano-stimulation on the activation of Wnt/β-catenin signalling pathway have been determined in different studies, employing TCF/LEF luciferase reporter transfected hMSCs or neuronal SH-SY5Y cells.^15,34,35^ To target the receptor, MNPs functionalized with an anti-Frizzled antibody or with the UM206 synthetic peptide, a specific ligand for the Frizzled receptor, can be used.

Another key mechanosensitive structures are ion channels, which play a crucial role in converting mechanical force into electrical and chemical signals.^92,93^ Stretching induced by magneto-mechanical stimulation can open ion channels and rise the cytosolic ion concentration, depolarizing the cell membrane; alternatively, the ions themselves can act as signalling messengers.^94^ There are many studies based on the use of magnetic actuators to target ion channels, especially TREK, TRP and Piezo. One example is the two-tandem pore potassium mechanosensitive ion channel TREK1, which is highly expressed in the cells of the nervous system, and in other non-neuronal tissues like osteoblasts.^95^ This protein can be stretch-activated through its extended loop region that acts as a tension spring. For this purpose, two approaches have been employed: (i) transfection into cells of a TREK1 receptor modified with an hexahistidine tag (His) that can be targeted with MNPs functionalized with an anti-His antibody;^10^ (ii) MNPs directly labelled with an anti-TREK1 antibody that can selectively bind to human bone marrow stromal cells (hBMSCs) and hMSCs that express this potassium channel.^82,96,97^ These studies used TREK1 as a target to stimulate osteogenesis.

Piezos are ionic channels that also play important roles in mechanotransduction, being found predominantly in non-neuronal cell types (Piezo1), or in mechanosensory cells such as hair follicles cells (Piezo2).^92^ Piezos are nonselective cation channels implicated in several physiological processes involving mechanical sensing, such as the sense of touch or cardiovascular homeostasis. Piezos are large proteins with 14–38 transmembrane domains unrelated to other known ion channel families. Although the exact mechanism of action is still unknown, it has been suggested that a lateral membrane tension can activate them.^92^ Interestingly, it has been described that Piezo1 can be directly and mechanically activated using MNPs and a magnetic field.^98^ In this case, cells were transfected with an engineered construct of this ion channel containing a bungarotoxin binding sequence, and incubated with biotinylated bungarotoxin, linking in this way the Piezo1 to the streptavidin-coated MNPs. Another approach to target Piezo1 is the use of anti-myc functionalized MNPs.^53^ To this purpose, Piezo1 modified with a Myc tag was genetically encoded into primary cortical neurons (see further details in section 4), demonstrating the feasibility to promote neuronal stimulation both *in vitro* and *in vivo*.

The TRP family cation channels participate in the sensing of different stimuli, such as heat, mechanical forces, light or pain among others.^99,100^ The TRP vanilloid (TRPV) subfamily has a tetrameric structure that conforms the ion conduction pore (usually for Ca^2+^), each monomer consisting of six transmembrane domains and three to six ankyrin repeats in the cytosolic N-terminal domain.^101^ Specifically, the thermosensitive TRPV1 (expressed in nervous cells) and TRPV4 (widely present in different types of cells) channels have been employed in magnetic neuromodulation using magnetic actuators.^13,102^ TRPV1 is a Ca^2+^-permeant channel and is activated mainly by heat (>42 °C), whereas TRPV4 is a nonselective cation channel that can be physically stimulated by mechanical forces and also by temperature.^99,103^ There are many examples of magnetic stimulation used to activate these channels, either mechanically or thermally. To this end, different strategies have been used, *e.g.* streptavidin-MNPs attached to the cell membrane containing TRPV1 channels through a specific binding with a biotinylated transmembrane protein previously transfected,^46^ green fluorescent protein (GFP)-tagged ferritin intracellularly synthetized and coupled inside the cell to a chimeric anti-GFP-TRPV1 transduced protein,^104^ or anti-His coated MNPs that directly interact with a transfected TRPV4 channel expressing an extracellular His tag,^105^ among others.

Finally, there are other ion channels that can be mechanically or thermally activated using magnetic actuators, like the N-type calcium ion channel or the temperature gated chloride channel anoctamin 1 (TMEM16A).^50,55,106^

## 4. Magnetomechanical stimulation

As magnetic actuators can act as stimulation probes, they have gained much attention for the interrogation of responses triggered by mechanical stimuli, generating a specific and remote mechanical stimulation on cell mechanosensitive receptors.^9,107^

One of the research areas in which magnetomechanical stimulation has been exploited the most is the control of stem cell fate for therapeutic purposes. For instance, Hughes *et al.* proposed one of the first examples of TREK1 ion channel opening using MNPs.^10^ Since then, many other works have described the use of magnetic actuators to target this ion channel.^82,96,97^ To this end, MNPs functionalized with an anti-TREK1 antibody or the RGD peptide (to target integrins) have been used for osteogenic and chondrogenic cell differentiation using *in vitro*, *in vivo* and *ex vivo* models.^82,96,97^ The first approach consisted in the labelling of hBMSCs with 250 nm silica MNPs functionalized with anti-TREK1 or RGD peptide.^82^ These cells were seeded alone or encapsulated into polysaccharide alginate/chitosan microcapsules and were tested *in vitro* or subcutaneously injected into mice. Thereafter, *in vitro* cultures or mice were stimulated using a magnetic force bioreactor,^108^ exposing them for 7 or 21 days to a pulsing magnetic field that moved up and down at 1 Hz frequency (1 hour daily). The estimated force under these conditions was 1–100 pN per particle. After the stimulation of TREK1 K^+^ channel, an increased, synthesis of proteoglycan and collagen was observed *in vitro*, and an enhanced production of extracellular matrix together with type-1 and type-2 collagen was observed *in vivo.* On the other side, the hBMSCs labelled with RGD-coated MNPs also showed an enhanced proteoglycan and collagen synthesis, together with an elevated extracellular matrix production and expression of type-1 and type-2 collagen *in vitro* and *in vivo.*

In a second approach, hMSCs were labelled with 300 nm commercial MNPs functionalized with the RGD peptide or an anti-TREK1 antibody that targeted an intracellular part of the ion channel.^96^ Labelled cells were microinjected on an *ex vivo* chick foetal femur and were magnetically stimulated with daily 1-hour sessions for 14 days at a frequency of 1 Hz and with a 25 mT magnetic field. This treatment generated a more extended mineralization on the epiphyseal injection site compared with the unlabelled control cells.^97^ The magnetic stimulation was performed using a commercially available vertical oscillating magnetic force bioreactor (MICA Biosystems), consisting on an incubation chamber, in which the samples or culture plates are situated above a magnetic array which oscillates vertically beneath with movement parameters controlled by a computer.^15^ Furthermore, the hMSCs were injected together with polymeric particles containing the bone morphogenic protein 2 (BMP2), demonstrating after a magnetic stimulation of 1-hour sessions for 14 days a positive synergistic effect in the mineralization of cells and surrounding tissues. In this way, a synergistical potential to combine the magnetic stimulation with pharmacological approaches is evidenced for applications such as the increase of bone formation. In a posterior study, a similar strategy was used, showing that mineralization was mainly mediated by the release of biological factors (cytokines and microvesicles) from the hMSCs, instead of the direct migration of the osteogenic cells.^97^

Besides ion channels, some receptors directly implicated in important mechanotransduction related pathways have been successfully targeted by magnetic actuators including Frizzled proteins, the principal receptors of Wnt ligands.^15^ As described before, this complex signalling pathway is implied in a multitude of cell responses, including osteogenic differentiation processes. To exploit this application, 250 nm dextran MNPs were coated with an anti-Frizzled antibody (Fz-MNPs) and targeted to hMSCs. Thereafter, hMSCs were magneto-stimulated by the commercial magnetic bioreactor (MICA Biosystems) previously described, using a magnetic field of 25–120 mT at a frequency of 0.9–1 Hz in 1- and 3-hour sessions. After magnetic stimulation of Fz-MNPs labelled cells, the nuclear localization of the Wnt intracellular messenger β-catenin increased when compared with controls without either Fz-MNPs or magnetic stimulation, showing a similar expression profile compared with the addition of recombinant Wnt3A protein. These results demonstrate that Frizzled receptors are mechanosensitive and the Wnt signalling pathway can be remotely activated *in vitro*, making possible to control stem cell fate for therapeutic purposes.^15^

**Fig. 6:**
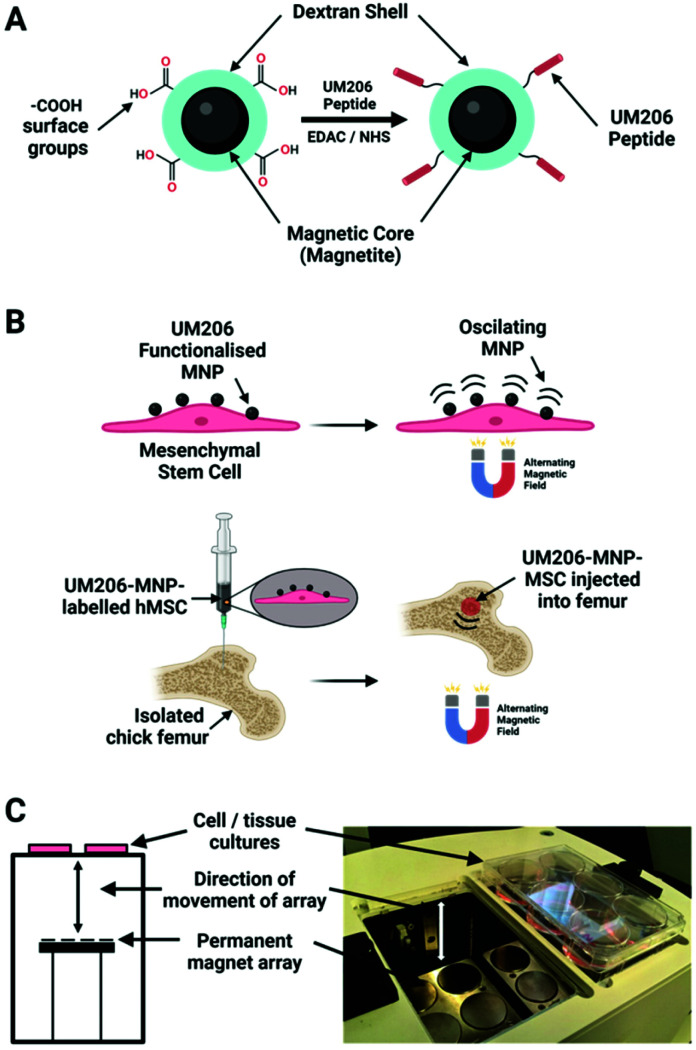
(A) 250 nm carboxy-dextran MNPs covalently functionalised with UM206 peptide by carbodiimide chemistry. (B) Human mesenchymal stem cells labelled with UM206-MNPs; the labelled cells were stimulated with oscillating magnetic fields or injected into foetal chick femurs before magnetic field stimulation. (C) Alternating magnetic fields were applied to samples using a magnetic bioreactor system (MICA Biosystems) consisting of arrays of permanent magnets situated beneath the samples. Adapted from *Nanomedicine: Nanotechnology, Biology and Medicine*, M. Rotherham, *et al.*, “Remote regulation of magnetic particle targeted Wnt signalling for bone tissue engineering”. vol. 14, pp. 173–184, copyright (2018), with permission from Elsevier. Figure adapted with *BioRender.com*.

In a later study, the same 250 nm dextran MNPs were conjugated to the synthetic peptide UM206 (UM206-MNPs), a ligand for the Frizzled receptor, and were targeted to hMSCs in order to activate the Wnt pathway (Fig. 6).^35^ After the magnetic stimulation of hMSCs labelled cells, it was reported a clustering of Frizzled receptors, β-catenin translocation and activation of TCF/LEF responsive transcription. Furthermore, hMSCs labelled with UM206-MNPs were injected in an *ex vitro* chick femur model showing an increased mineralization together with a synergistic effect in presence of microparticles containing BMP2.

As mentioned earlier, the Wnt pathway has also been related to neurogenesis processes, being able to regulate dopaminergic progenitor cell differentiation in neuronal development. Therefore, it is an interesting target for diseases like Parkinson. The UM206-MNPs previously described were also targeted to Frizzled receptors present on SH-SY5Y neural cells with the capacity to differentiate into neuron-like cells when cultured with neurotrophic factors, showing a neuronal morphology and expressing typical neuronal markers.^34^ This time, the UM206-MNPs labelled cells were magnetically stimulated with ≥25 mT magnetic fields in 1–3 hours sessions at frequency of 1 Hz using the previously described commercial magnetic reactor (MICA Biosystems).^15,34^ After magnetic stimulation, cells labelled with both peptide variants showed an increased β-catenin translocation compared with treatments without magnetic stimulation. An enhanced activation of TCF/LEF responsive transcription was also observed compared with controls without UM206-MNPs or without magnetic stimulation.^34^ In addition, dopaminergic differentiation was also evaluated by culturing SH-SY5Y cells on dopaminergic induction media containing phorbol 12-myristate 13-acetate (PMA). The same magnetic stimulation conditions were applied as described before for 4 days. After stimulation, the expression of key dopaminergic markers was determined, demonstrating an enhanced expression of these markers on UM206-MNPs labelled cells after magnetic stimulation compared with those without stimulation. These results demonstrate that remote activation of Wnt signalling can produce an increase of dopaminergic differentiation of SH-SY5Y in presence of PMA.^34^

**Fig. 7:**
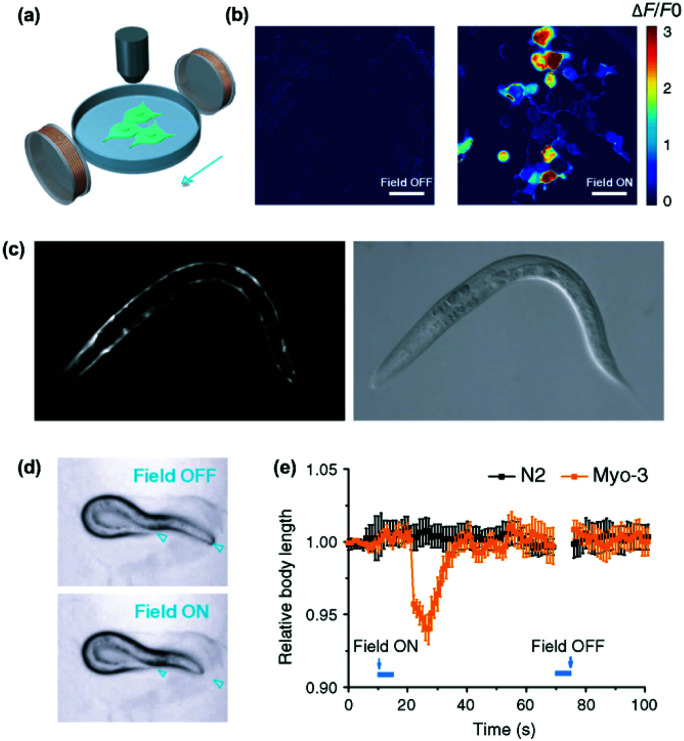
Magnetogenetic activation of HEK-293 cells and control of behavioural responses in *C. elegans* by remote magnetic stimulation. (a) Schematic of magnetic stimulation of MAR co-transfected HEK-293 cells together with the calcium indicator GCaMP6s with a pair of electrical coils. (b) Change of fluorescence intensity before and after remote magnetic stimulation. (c) Epifluorescence (left) and bright field (right) images of transgenic *C. elegans* expressing MAR under the promoter *myo-3*. (d) Body contraction before (top) and after magnetic field application (bottom). (e) Measurements of body length before and after magnetic field application at different time points. While *C. elegans* expressing MAR under myo-3 promoter showed body contraction (orange trace), N2 wild type showed no obvious change of body length after magnetic stimulation. Reprinted from Long, X. *et al.*, Magnetogenetics: Remote Non-Invasive Magnetic Activation of Neuronal Activity with a Magnetoreceptor. *Sci. Bull*., 2015, **60**(24), 2107–2119 (article distributed under the terms of the Creative Commons CC-BY license).

Non-invasive modulation of neuronal activity is a burgeoning research field, in which magnetogenetics has gained great attention. In order to trigger neuronal activity, Long *et al.* used a pigeon homologue of human iron sulphur cluster assembly protein 1 expected to bind to the cellular membrane as a magnetoreceptor (MAR).^109^ Human embryonic kidney cells (HEK-293) transfected with MAR were stimulated with a homemade magnetic generator, composed by a pair of electromagnetic coils, able to produce a maximum strength of 2.5 mT on the dish edge and 1 mT at the dish centre (Fig. 7a and b). A minimum strength of 0.3 mT was required for detecting a calcium influx after membrane depolarizing. Similar results were obtained using static magnetic bars and primary cultured rat hippocampal neurons instead of HEK-293 cells. The authors also investigated the possibility of activating neurons changing the magnetic field direction, that is, generating magnetic fields only in the X or the Y direction. However, no obvious correlation between the magnetic field direction and the triggered response was found. To test if MAR could trigger this activation *in vivo*, a transgenic *Caenorhabditis elegans* worm expressing MAR only in the muscle cells was created (by expressing MAR under the control of the promoter *myo-3*). After applying the magnetic field, the transgenic nematode showed muscle contractions and shrinkage of body length (Fig. 7c–e).

Similarly, Wheeler *et al.* generated a protein called Magneto 2.0, consisting of TRPV4 fused to a gene encoding two subunits of the ferritin, and bearing a plasma membrane trafficking signal.^13^ The rationale behind this design was the possibility to mechanically open TRPV4 using the magnetic torque of the ferritin attached to it under the application of a magnetic field. After applying a 50 mT magnetic field delivered by an electromagnet, intracellular calcium increased in HEK-293 cells expressing Magneto 2.0, but not in other control cells. Calcium transients were dependent on TRPV4 as revealed by treating the cells with an inhibitor. Importantly, Magneto 2.0 could also trigger the response of sensory neurons in zebrafish and of striatal dopamine 1-expressing neurons in mice. In the case of mice, these neurons are involved in reinforcing behaviour. When animals were placed in a chamber with two arms, mice showed preference for the chamber where magnetic fields were applied (magnetic field gradient of 250–50 mT) instead of the unmagnetized arm of the chamber. Similarly, ferritin tethered to TRPV1 has also been used to activate neurons, either using an alternating or a gradient magnetic field (see section 5).^104,110^

**Fig. 8:**
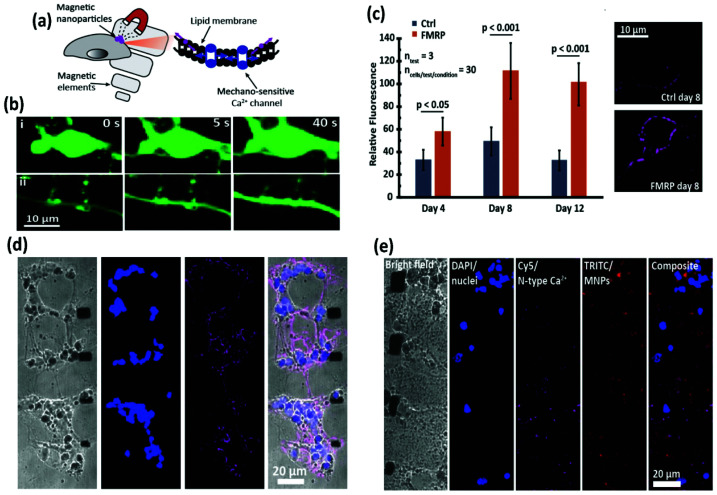
Scheme of magnetic technique and modulation of excitatory N-type Ca^2+^ channels in an FXS neural network model following magnetic stimulation. (a) Schematic of the technique. Neurons grown on substrates that produce high local field gradients are stimulated with MNPs and a permanent magnet to induce calcium influx. (b) Fluorescence microscopy images showing an increase in calcium both in (i) the cell body and (ii) the axonal boutons. (c) FXS model neurons (FMRP) express more N-type calcium ion channels than normal neurons. With age, however, there is a decrease in N-type calcium ion channel expression. Immunostaining of N-type calcium ion channels in the FXS model neurons (top) and control neurons (bottom). (d) FXS model neurons and (e) FXS model neurons after magnetic chronic stimulation. A decrease in N-type calcium ion channel florescent intensity is observed in (e). Reprinted with permission from Tay, A., & Di Carlo, D. (2017). Magnetic Nanoparticle-Based Mechanical Stimulation for Restoration of Mechano-Sensitive Ion Channel Equilibrium in Neural Networks. *Nano letters*, **17**(2), 886–892. Copyright (2017) American Chemical Society.

Starch-coated MNPs (100 nm) have been used for unspecific attachment to the cell membrane for neuromodulation and restoration of ion channel disequilibrium in neural networks.^55^ To this end, neurons were dissociated from the cortices of E18 Sprague Dawley rats and were kept for two weeks in culture, until mature synapse formation was reached. The neurons were grown on microfabricated substrates that produced high local magnetic field gradients, incubated with MNPs and stimulated using a neodymium magnet providing a magnetic field of 150 mT (maximum strength) (Fig. 8). In this way, the local magnetic stimulation induced calcium influx through mechanical activation of mechanosensitive N-type excitatory Ca^2+^ channels. However, the involvement of other mechanosensitive channels such as TRPV4, Piezo1 and NMDA could not be ruled out. Besides the ability to modulate N-type Ca^2+^ channels, a fragile X syndrome (FXS) neuron network model was used.^55^ These neurons expressed the fragile X mental retardation protein, which increases the density of N-type Ca^2+^ channels, leading to hyperexcitability. Interestingly, neuron networks are known to regulate the equilibrium between excitatory (*i.e.*, N-type Ca^2+^ channels) and inhibitory receptors (*i.e.*, gamma-aminobutyric acid-GABA) in order to keep homeostasis. After chronic mechanical stimulation (4 days) of the FXS neurons containing MNPs, the expression of N-type ion channels decreased to a similar level to that of control neurons, demonstrating the possibility to regulate the equilibrium of receptors through magnetogenetic actuation.

**Fig. 9:**
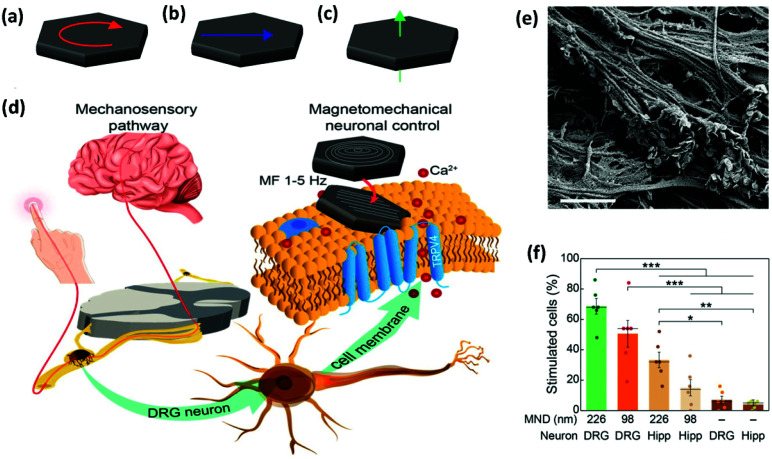
Magnetomechanical stimulation of MND-decorated DRG neurons allows for remote activation of Ca^2+^ influx. (a–c) Schematic representation of the three possible configurations of magnetic spins in MNDs: (a) vortex, (b) in-plane, and (c) out-of-plane (see section 2.2.1. for further details). (d) DRGs relay mechanosensory information to the spinal cord. DRG explants were incubated with MNDs and stimulated using slowly varying (≤5 Hz) magnetic fields. MNDs were then magnetized in a direction compatible with their easy axes (on the plane of the disk), generating force on ion channels and the concomitant mechanical torque and Ca^2+^ influx. (e) SEM image of the DRG explant culture surface incubated with individual MNDs that can be observed on the surface. Scale bar = 2 μm. (f) Comparison of the efficacy (percentage of stimulated cells in calcium imaging) of magnetomechanical stimulation for 226 and 98 nm diameter MNDs on DRGs or hippocampal neurons (Hipp). A two-way ANOVA was conducted on the influence of culture (DRG or Hipp) and MND type (226, 98 nm or none), being significant the main effects for culture type and the interaction. Reprinted with permission from Gregurec, D., *et al.*, “Magnetic Vortex Nanodiscs Enable Remote Magnetomechanical Neural Stimulation”. *ACS Nano*, 2020, **14**(7), 8036–8045. Copyright (2020) American Chemical Society.

In a different set of experiments, MNDs of different sizes were smartly designed to exert a mechanical torque on neuronal receptors, triggering a calcium influx (see section 2.2.1 for further details on the MNDs).^52^ Root ganglia explants (DRGs), which contain sensory neurons, were incubated with MNDs and stimulated slowly varying (≤5 Hz) magnetic fields in three periods of 10 s, observing a higher calcium ion influx for larger MNDs, when compared to smaller ones (Fig. 9). The same experiment was performed using hippocampal neurons, which have limited mechanosensitivity, observing negligible or smaller responses. The addition of different ion channel inhibitors suggested that Piezo, TRPV4 and TREK could be all involved in the response to mechanical stimuli. On the contrary, sodium channels and gap junctions present in neurons and glia, respectively, were not involved in the response.

**Fig. 10:**
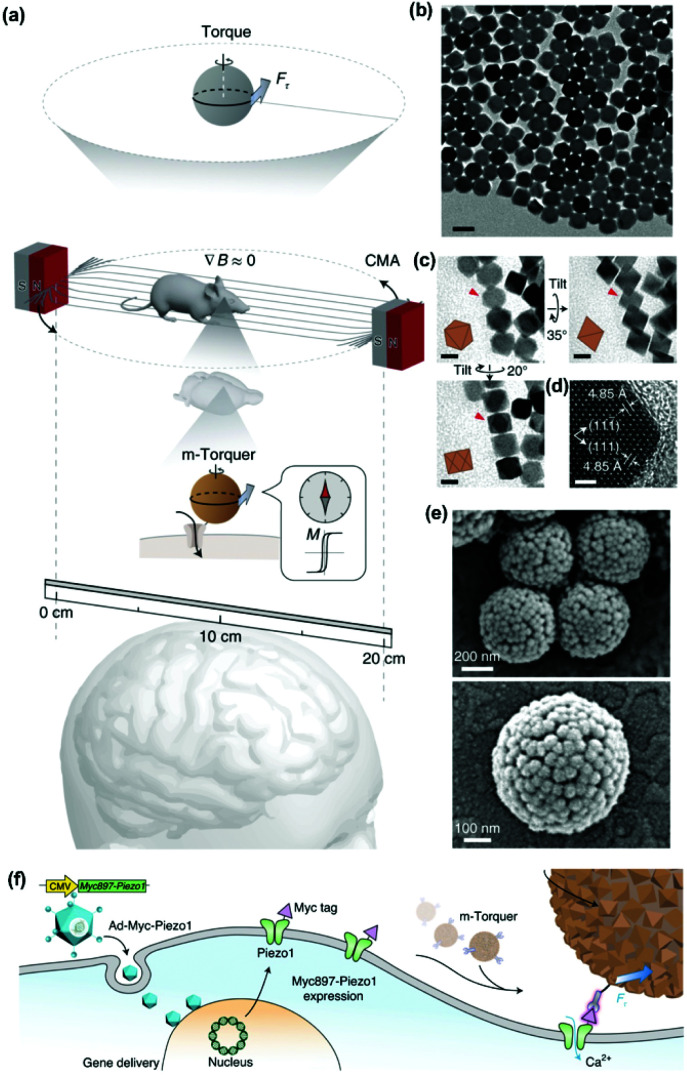
Magnetomechanical gating of Piezo1 ion channel in cultured neurons with m-Torquer system. (a) Schematic of the nanoscale m-Torquer system. The m-Torquer system is composed of a rotating uniform magnetic field (∇*B* ≈ 0) generated by a circular magnet array (CMA) and a nanoparticle m-Torquer generator. The working distance of the m-Torquer system can be increased to tens of centimetres with the potential application. (b) TEM image of 25 nm octahedral MNPs. Scale bar, 50 nm. (c) TEM tilting analysis shows the octahedral shape of nanoparticles. Scale bars, 20 nm. (d) High-resolution TEM image of the MNPs. Scale bar, 2 nm. (e) SEM images of m-Torquers composed of assembled monolayer octahedral nanoparticles on a spherical support *via* click chemistry. Scale bar, 200 nm and 100 nm. (f) Schematic of genetic encoding of Piezo1 by Ad-Piezo1 with human cytomegalovirus (CMV) promotor and its magnetomechanical gating with specifically targeted m-Torquer with Myc antibody. Reprinted by permission from Springer Nature, *Nature Materials*, “Non-Contact Long-Range Magnetic Stimulation of Mechanosensitive Ion Channels in Freely Moving Animals”, Jung-uk Lee *et al.*, Copyright (2021).

One of the most exciting examples of *in vivo* magnetomechanical neuronal control has been recently described by Lee *et al.*^53^ The authors generated a set of magnetic tools coined as m-Torquer, consisting of a nanoscale magnetic torque actuator for untethered and fast neuromodulation at a long working range. This toolkit is composed of 25 nm octahedral MNPs assembled on a 500 nm spherical polystyrene support in order to exhibit extremely high magnetic moments and a rotating circular magnet array (Fig. 10). The magnet array is designed to provide a rotating uniform magnetic field with a working range of ∼70 cm, generating pN-scale torque forces to the cells. *In vitro*, mouse primary cortical neurons were infected by an adenovirus containing the mechanically sensitive residue 897 of Piezo1 modified with a myc tag (Ad-Piezo1), allowing a density of four Piezo1 channels per μm^2^. m-Torquer were functionalized with an anti-myc antibody in order to bound to Piezo1. Stimulation of Piezo-bound m-Torquer in neurons with a rotating magnetic field at 0.5 Hz resulted in an increased expression of c-Fos mRNA, a reporter gene for neuronal activation by calcium influx, while control groups did not show overexpression of c-Fos. Using X-Rhod-1 Ca^2+^, a fluorescent dye indicator of calcium influx in live cells, an increase in intracellular calcium influx was observed when applying the magnetic field, while control groups did not show calcium responses. For *in vivo* experiments, mice were injected with the Ad-Piezo1 into the motor cortex of the brain; after four weeks, m-Torquer system functionalized with anti-myc were also injected only in the right hemisphere and stimulated with the rotating circular magnet array at 0.5 Hz. A homogeneous expression of Piezo1 was found in the motor cortex and neuronal activation in the region of interest was confirmed by c-Fos staining. In contrast, c-Fos signals were not generated when using m-Torquer not functionalized with anti-myc antibody, demonstrating the selective character of the neuromodulation.

## 5. Magnetothermal stimulation

Magnetothermal stimulation typically relies on the activation of temperature-sensitive transmembrane proteins, with the TRPV1, also known as the capsaicin receptor, being the most common one.

**Fig. 11:**
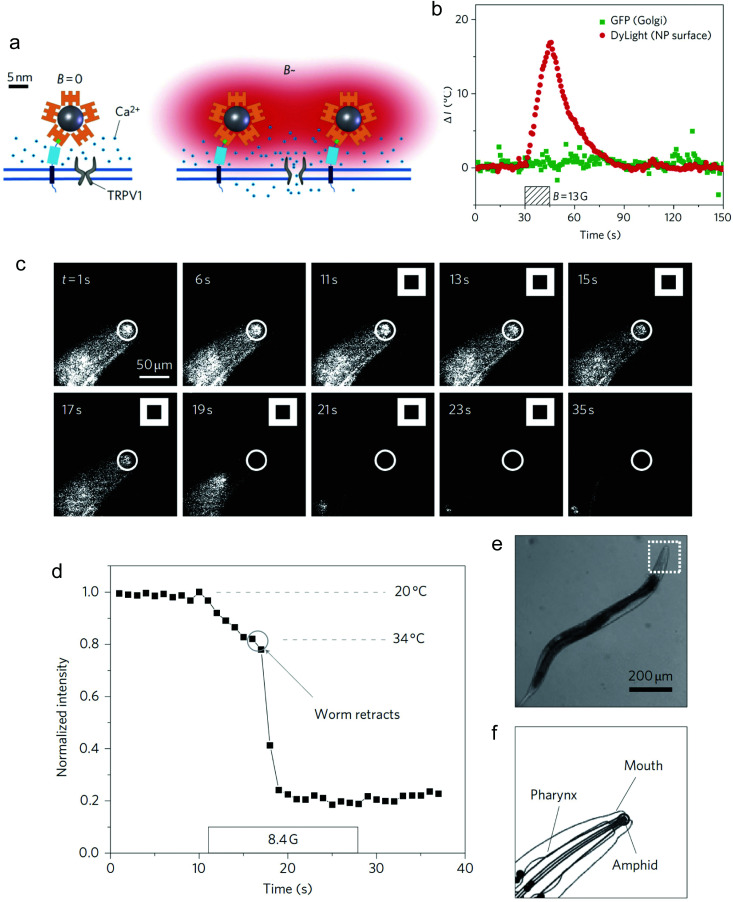
(a) Principle of TRPV1 opening post-thermal stimulation with MNPs and AC fields. Streptavidin-DyLight549-coated MNPs bind to the biotinylated membrane protein AP-CFP-TM. (b) Variation of the local temperature at the plasma membrane (red) and the Golgi apparatus (green), measured by the changes in the fluorescence intensity of DyLight549 (membrane) and Golgi-targeted GFP (Golgi apparatus), respectively. (c) Fluorescence image sequence of the head region of a *C. elegans* worm labelled with MNPs. The white squares indicate the retraction movement of the animals as a consequence of the increase in temperature upon the application of the AC field (thermal avoidance). (d) Time-dependence of the fluorescence intensity and temperature of the amphid region of the worm. (e) Bright-field image of the worm, indicating the head region labelled with MNPs. (f) Schematic drawing of the head region and its structures. Reprinted by permission from Springer Nature, *Nature Nanotechnology*, “Remote control of ion channels and neurons through magnetic-field heating of nanoparticles”, H. Huang, S. Delikanli, H. Zeng, D. M. Ferkey, A. Pralle, copyright (2010).

Neuronal activation is one of the most explored applications of magnetothermal stimulation, with several proof-of-concept experiments described so far. In one of the first examples of use of localised heating produced by MNPs for remote cellular stimulation, Huang *et al.* reported the thermal activation of temperature-sensitive ion channels TRPV1 with 6 nm manganese ferrite (MnFe_2_O_4_) nanoparticles.^46^ The nanoparticles were coated with streptavidin and modified with DyLight549 fluorophore to enable the temperature measurement in the vicinity of the nanoparticle surface by monitoring the changes in the fluorescence intensity of the probe (Fig. 11a). While a significant increase in the local temperature could be estimated when the MNPs were exposed to a radiofrequency (RF) magnetic field (40 MHz, 0.67 kA m^−1^), no appreciable bulk heating occurred. Moreover, in HEK-293 cells engineered to express Golgi-targeted GFP and a biotinylated cell membrane protein to bind the streptavidin-coated MNPs, the application of the magnetic field led to a decrease in the fluorescence intensity of the cell surface DyLight549 fluorophore, pointing out to an increase of the temperature of more than 15 °C in 15 s (Fig. 11b). Conversely, the GFP fluorescence intensity in the cytoplasm remained practically constant, demonstrating that the heating was specifically localised on the plasma membrane. The remote control of neuronal TRPV1 channels was also demonstrated *in vivo*, triggering behavioural responses (thermal avoidance) in *C. elegans* worms labelled with nanoparticles and subjected to the AC field (Fig. 11c–f).

**Fig. 12:**
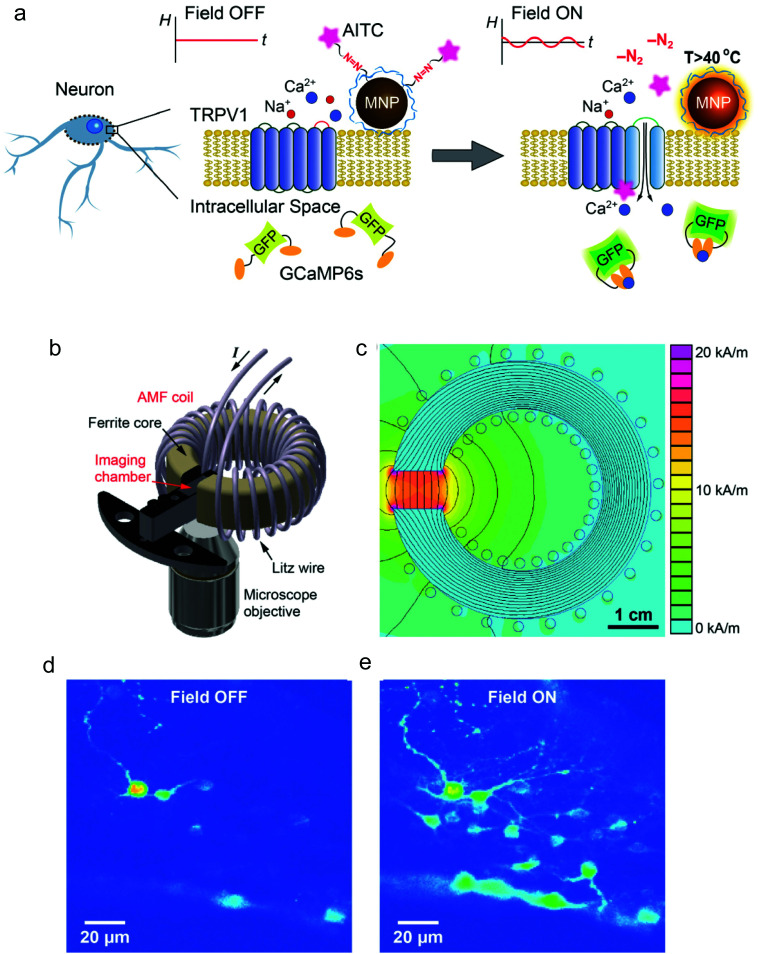
(a) Principle of the magnetothermal activation approach using AITC bound to MNP surface through a thermolabile linker, which can be cleaved upon exposure to the AMF. (b) 3D scheme of the experimental setup, depicting the AMF coil and the sample chamber. (c) Plot of the field amplitude as a function of the position (finite element simulation of the coil cross section). (d) and (e) Fluorescence images of neurons expressing TRPV1 and GCaMP6s and treated with AITC-modified MNPs before (d) and after 15 s of exposure to the AMF (e). The increase in the fluorescence intensity observed in (e) indicates Ca^2+^ influx. Reprinted with permission from G. Romero, *et al., Adv. Funct. Mater.*, **26**, 6471–6478, “Localized Excitation of Neural Activity *via* Rapid Magnetothermal Drug Release”, copyright (2016), WILEY-VCH Verlag GmbH & Co. KGaA, Weinheim.

Using 22 nm magnetite nanoparticles coated with polyethylene glycol (PEG) for improved biocompatibility, Anikeevás group demonstrated the TRPV1 activation in hippocampal neurons *in vitro* and of deep brain neurons in ventral tegmental area (VTA) of mice brains upon the application of the AC magnetic field (500 kHz, 15 kA m^−1^).^111^ The *in vivo* effect of the thermal stimulation was assessed by quantifying the expression of the c-Fos gene, an indirect marker of neuronal activity.^112^ Interestingly, VTA neurons could also be activated one month post-MNP injection, suggesting the potential long-term utility of magnetogenetics for remote stimulation of brain activity. The same group reported neuronal excitation following a slightly different approach, namely the use of allyl isothiocyanate (AITC) as agonist of TRPV1 channels.^37^ AITC was covalently bound to the surface of 25 nm iron oxide MNPs *via* thermolabile azo linkers and released upon the application of an AMF (500 kHz, 15 kA m^−1^), triggering Ca^2+^ influx, which was monitored using the fluorescence signal of GCaMP6s, a genetically encoded Ca^2+^ indicator (Fig. 12). Nevertheless, in these two studies the MNPs lacked a direct targeting to the TRPV1 channels; for *in vitro* studies, their attachment to the cell membrane relied on surface functionalisation with PEG to prevent internalisation^111^ and with poly(ethyleneimine) to enable membrane binding,^37^ while for *in vivo* experiments MNPs were injected in the brain area in which the TRPV1 expressing neurons were located.

**Fig. 13:**
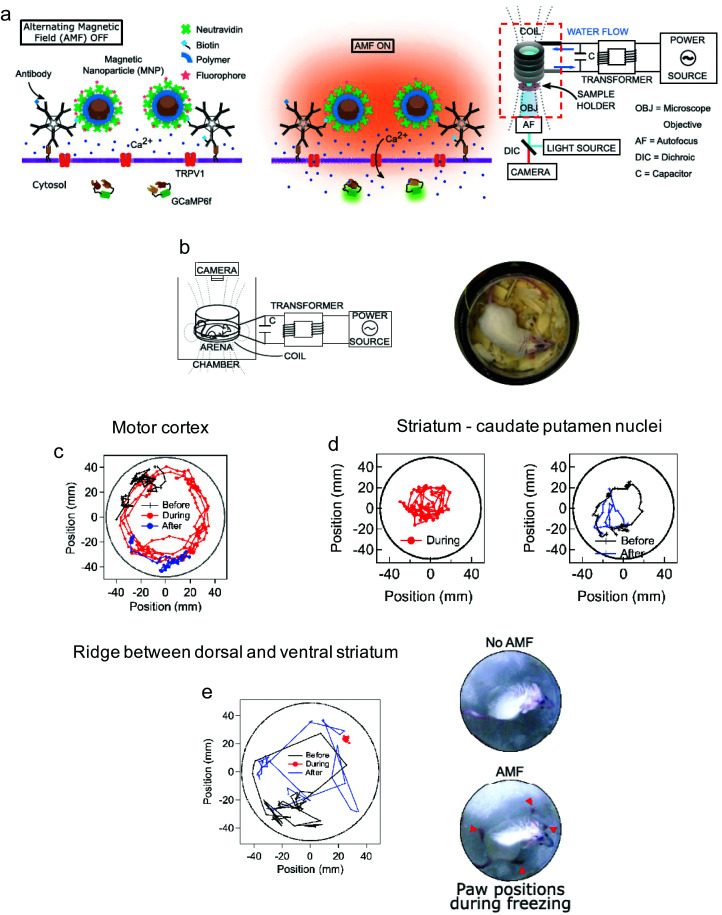
(a) Left: Magnetothermal activation of thermosensitive TRPV1 ion channels with MNPs bound to the cell membrane. Right: experimental setup for *in vitro* experiments, combining the AMF application with fluorescence microscopy. (b) Experimental setup for *in vivo* magnetothermal stimulation of motion behaviour of awake mice and photograph of the animal in the observation zone (depicted as arena). (c) 1 min-long trajectories of a mouse stimulated in the motor cortex before (black), during (red), and after (blue) field application, showing a fast movement around the arena. (d) 1 min-long trajectories of a mouse stimulated in the caudate putamen nuclei. In this case, the animal remained near the centre of the arena and rotated around its body axis. (e) 1 min-long trajectories of a mouse stimulated near the ridge between dorsal and ventral striatum, showing the freezing of gait. The “frozen” position of the mouse paws is shown in the right bottom photograph. Reprinted from R. Munshi, *et al.*, “Magnetothermal genetic deep brain stimulation of motor behaviors in awake, freely moving mice”, *eLife*, 2017, **6**, e27069, copyright 2017, (article distributed under the terms of Creative Commons Attribution 4.0 International License).

Going one step further, Munshi *et al.* demonstrated the magnetothermal activation of three areas in the brain of awake mice, each of them being responsible of controlling a different motor behaviour.^58^ The MNPs used in this study had an 8 nm Co ferrite core surrounded by a 2.25 nm Mn ferrite shell and a ∼5 nm polymer coating (dodecyl-grafted-poly-(isobutylene-*alt*-maleic-anhydride), PMA) to ensure colloidal stability in physiologically relevant media. The MNPs were further functionalised with neutravidin to allow specific binding to biotin-modified A2B5 antibody targeting neuronal glycosylated membrane proteins (Fig. 13a). Stimulation of the motor cortex neurons overexpressing TRPV1 in mice treated with the MNPs and exposed to an AMF (570 kHz, 7.5 kA m^−1^) elicited fast movement of the animals around the arena (Fig. 13b and c); this stimulated ambulation persisted over the course of two days. The same approach was used to stimulate deeper brain regions. In the striatum, activation of caudate putamen nuclei led to a rotational movement of the mice around their bodies (Fig. 13d), while stimulation of deeper areas (the ridge between dorsal and ventral striatum) resulted in a motion inhibitory response, with the animals having the four paws frozen in place but maintaining the ability to rotate their heads (Fig. 13e). Since in this work the MNPs were attached to the cell membranes *via* specific antibody-receptor interactions, a more efficient and robust stimulation could be achieved with a lower dose of nanoparticles (the authors report a 200-fold lower amount than the one used by Anikeeva's group^111^). In a similar antibody-receptor targeting approach reported by the same group, magnetothermal actuation was explored as a tool for neuronal silencing by targeting the temperature gated chloride channel TMEM16A.^50^ Core–shell MNPs (13 nm diameter, Mn ferrite core and Co ferrite shell) coated with PMA and modified with neutravidin were targeted to the cell membrane of rat neurons co-transfected to express TMEM16A and the cytosolic calcium indicator protein GCaMP6f. Upon exposure to the magnetic field (412.5 kHz, 28.9 kA m^−1^), only TMEM16A^+^/MNP^+^ neurons showed significant changes in their firing rates.

Besides neuronal stimulation, the magnetothermal activation of the heat-sensitive channel TRPV1 has been also addressed for remote regulation of protein production. Stanley *et al.* used the calcium influx post-TRPV1 activation to stimulate the synthesis of insulin *in vivo* in mice.^39^ Iron oxide nanoparticles (20 nm) were modified with anti-His antibodies to specifically bind a TRPV1 channel engineered to express an extracellular hexahistidine tag (TRPV1^His^). Mice bearing tumours derived from neuroendocrine PC12 cells transfected to express TRPV1^His^ and a calcium-dependent insulin construct were treated with MNPs by intratumoural injection and subjected to a radiofrequency field (4 kA m^−1^, 465 kHz). The treatment led to a significant increase of plasma insulin, with the subsequent decrease of the blood glucose levels.

**Fig. 14:**
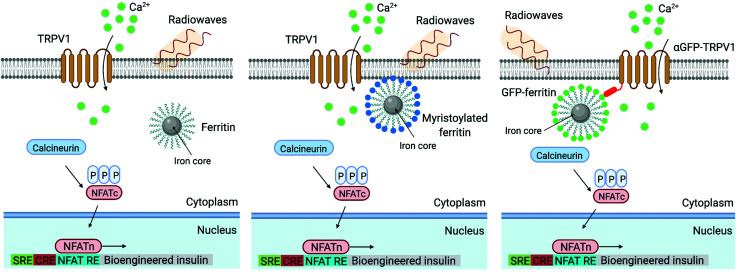
Schematic depiction of three alternate locations of genetically encoded ferritin nanoparticles used to trigger the TRPV1 opening: cytoplasmic (left), membrane-tethered (middle) and channel-associated (right). Adapted by permission from Springer Nature, *Nature Medicine*, “Remote regulation of glucose homeostasis in mice using genetically encoded nanoparticles”, S. A. Stanley, J. Sauer, R. S. Kane, J. S. Dordick, J. M. Friedman, copyright (2015). Figure created with *BioRender.com*.

In all the examples described so far in this section, the thermal stimulation was based on the use of synthetic MNPs. This approach requires biocompatible and suitably functionalised MNPs, able to target relevant cell surface biomarkers; in some instances, it could also require a re-injection of the nanoparticles at a later stage in order to achieve a long-term effect. As an alternative, Stanley *et al.* proposed the use of endogenous, intracellularly synthesised ferritin nanoparticles to modulate *in vivo* insulin gene expression^110^ or to activate glucose-sensing neurons^104^ upon exposure to AC fields. Interestingly, genetically encoded ferritin nanoparticles with three different intracellular locations (cytoplasmic, membrane-tethered and TRPV1-associated, see Fig. 14) induced different levels of insulin gene expression, the highest ones being achieved with the membrane-linked and the TRPV1-associated constructs.^110^

## 6. Other types of magnetic actuator-mediated stimulation

A third mechanism of remote manipulation of cellular signals with magnetic actuators involves the controlled clustering of membrane receptors^11,12,48,113–116^ or modulation of the intracellular distribution of specific molecules.^117–119^

In a pioneering study, the group of Ingber demonstrated the possibility of using a magnetic switch to trigger a biochemical signalling mechanism conventionally activated by multivalent ligand binding.^11^ RBL-2H3 mast cells were modified with IgE antibodies directed against the dinitrophenyl (DNP) antigen, so that the cells would display these antibodies bound to FcεRI receptors. 30 nm MNPs coated with DNP ligands could attach to cell surface IgE-FcεRI, resulting in receptor clustering and activation of an intracellular signalling response characterized by an increase in intracellular calcium concentration. Only when the antigen density of each MNP was high enough (30 antigens per MNP), activation was detected. An external magnetic field provided by an electromagnetic needle was used to trigger receptor clustering when using MNPs functionalized with only one DNP ligand per MNP. In this case MNPs reversibly aggregated through dipole–dipole interactions.

**Fig. 15:**
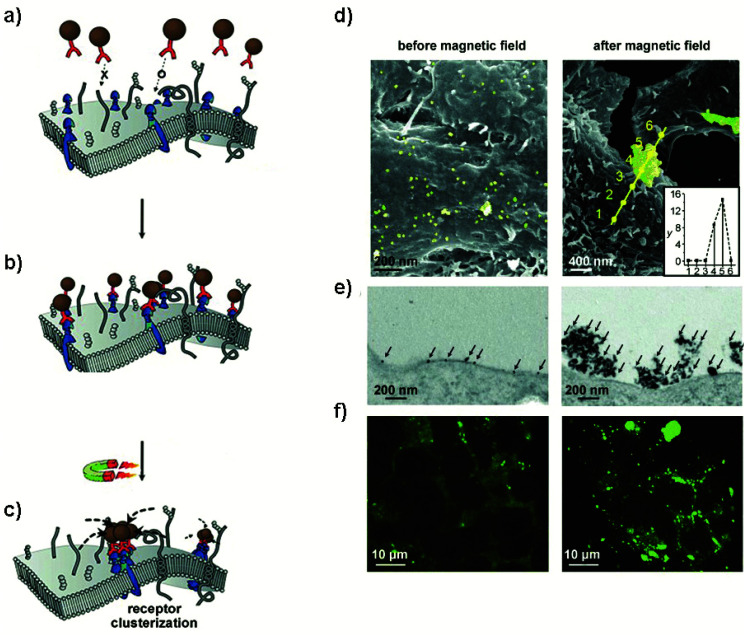
Clustering of Tie2 receptors using MNPs. (a–c) Targeting and magnetic manipulation of Ab-Zn-MNPs. (a and b) MNPs modified with a monoclonal antibody against Tie2 receptors, selectively bind to them. (c) After applying an external magnetic field, the Ab-Zn-MNPs form aggregates, inducing the clustering of Tie2 receptors. (d–f) Magnetism-induced aggregation of Ab-Zn-MNPs on the cell surface. (d) SEM images of the MNPs on the cell surface before and after magnetic field application. Ab-Zn-MNPs are shown in yellow for clear visibility. MNP aggregates after applying the magnetic field were analysed using EDX. A high Fe content was found on the aggregates (inset in the image on the right). (e) TEM images of the MNPs (indicated by arrows) before and after magnetic field application. (f) Fluorescence confocal microscopy images of MNPs before and after application of a magnetic field. Reproduced with permission from Lee, J.-H. *et al.*, *Angewandte Chemie International Edition*, **49**, 5698–5702 (2010), “Artificial Control of Cell Signaling and Growth by Magnetic Nanoparticles” copyright (2010) Wiley-VCH Verlag GmbH & Co. KGaA.

Likewise, Lee *et al.* used 15 nm Zn^2+^-doped ferrite MNPs conjugated with a monoclonal antibody to target Tie2 receptors (Ab-Zn-NPs) (Fig. 15).^48^ Conventionally, one angiopoietin molecule binds to cluster several Tie2 receptors, triggering intracellular signalling pathways that can participate in the angiogenesis process. Artificial clustering of Tie2 receptors was performed using Ab-Zn-NPs and two permanent NdFeB magnets that exerted a homogeneous DC magnetic field of approximately 150 mT. In the case of epidermal growth factor receptors (EGFR), receptor clustering was achieved using MNPs coated with antibodies against EGFR or MNPs directly targeted to another site of the receptor ectodomain.^114^ A short (three minutes) exposure to a static magnetic field of 376 mT was enough to fully activate the EGFR signalling on cells.

**Fig. 16:**
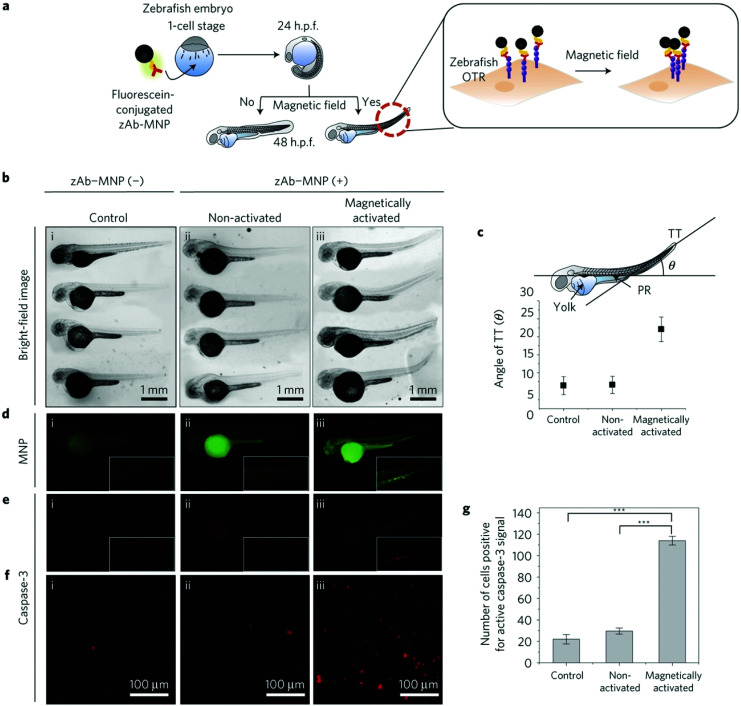
*In vivo* magnetic apoptosis signalling for zebrafish. (a) Scheme of the magnetogenetic experiment in zebrafish. Ab–MNPs functionalized with fluorescein are injected into yolk of embryo at one-cell stage to label OTR. At 24 h post-fertilization (h. p. f.), zebrafish are divided into two groups (control and activated with magnetic field). (b) Bright-field microscope images of three groups of animals, (i) control, (ii) incubated with Ab-MNPs and non-activated and (iii) incubated with Ab-MNPs and magnetically activated. The latter group shows morphological alterations in the tail region when compared with other groups. (c) Quantitative analysis on morphological alterations (tail bending) after applying magnetic hyperthermia. The angle between the line on the pronephros (PR) and the line of tail tip (TT) is measured for each group. (d–f) Fluorescence images of zebrafish showing the activation of caspase-3 after magnetic manipulation: Ab–MNPs is shown in green, whereas active caspase-3 is shown in red. Red fluorescence is only observed in the tail region of group iii, treated with MNPs and stimulated with the magnetic field. (f) Magnified images of active caspase-3. (g) Graph quantifying the number of cells positive for caspase-3 activity (***P < 0.001). Reprinted by permission from Springer Nature, *Nature Materials*, “A magnetic switch for the control of cell death signalling in *in vitro* and *in vivo* systems”, Mi Hyeon Cho *et al.*, copyright (2012).

The possibility of developing magnetic switches to cluster receptors has also been demonstrated *in vivo*. For example, 15 nm Zn_0.4_Fe_2.6_O_4_ MNPs have been used to promote apoptosis signalling pathways both *in vitro* and *in vivo*. To this end, antibody-conjugated MNPs (Ab-MNPs) were injected into the yolk of zebrafish embryos to target the ovarian tumour necrosis factor receptor (OTR) (Fig. 16).^12^ After applying a uniform DC magnetic field (500 mT) for 24 h, apoptosis signalling pathways were activated. As a consequence, activation of caspase-3 and morphological alterations in the tail region were observed for this group of animals when compared with control ones (Fig. 16).

Similarly, the possibility to stimulate both extrinsic and intrinsic apoptotic pathways to overcome cancer multidrug resistance (MDR) using a magnetic switch and controlled receptor aggregation has been described.^113^ In this case, 15 nm Zn_0.4_Fe_2.6_O_4_ MNPs were modified with doxorubicin (Dox, an anticancer drug) using a cleavable disulphide linker and targeted to the death receptor 4 (DR4) by monoclonal antibodies. An MDR tumour xenograft mouse model was used, exposing the animals to a static magnetic field of 500 mT for 3 h. Due to the MDR characteristics of the tumour model, 14 days after the treatment Dox alone could not inhibit the tumour growth, while the use of the magnetic switch completely removed it within 10 days.

Receptor clustering by MNPs can also be used to stimulate T cell activation^115,116^ and antitumour activity *in vivo*.^115^ Perica *et al.* used 50–100 nm iron-dextran NPs functionalized with a chimeric major histocompatibility complex-Ig dimer and anti-CD28 antibodies to activate T cell receptor (TCR) clustering and T cell proliferation.^115^ To this end, two neodymium magnets generating a maximum field strength of 200 mT were used. Transferring magnetic field activated T cells to mice bearing a poorly immunogenic tumour (B16) led to inhibition of tumour growth and increase of survival. Interestingly, magnetic microparticles in the presence of a magnetic field were not able to enhance clustering of receptors due to their large size compared to TCR nanoclusters.

**Fig. 17:**
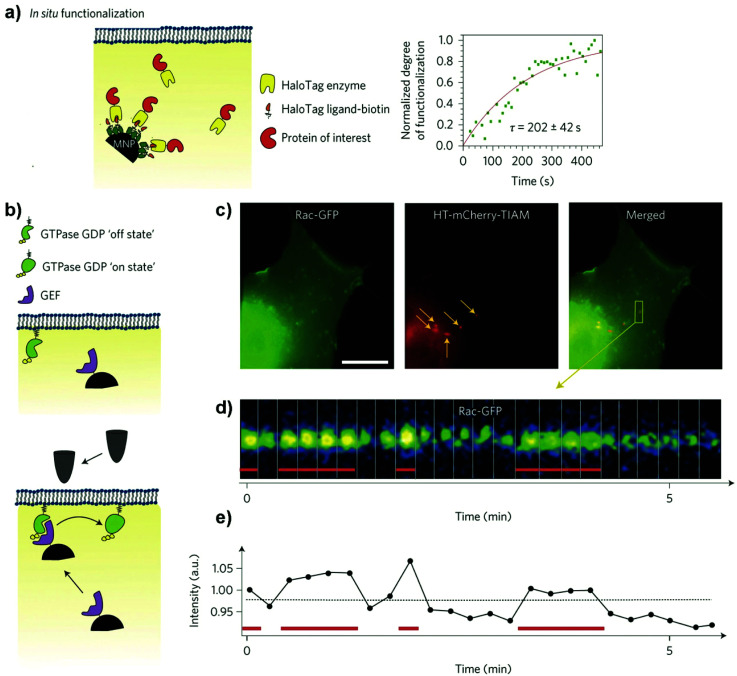
(a) *In situ* functionalization scheme: Left: streptavidin-coated MNPs functionalized with a biotinylated -HaloTag Ligand (HTL) are microinjected into cells expressing the protein of interest fused to the HaloTag. The HaloTag binds irreversibly to its ligand, recruiting the protein of interest to the MNP. Right: kinetics of HaloTag–mCherry binding to HTL-MNPs. (b) Remote control of Rho-GTPase signalling at the plasma membrane: Top: GEF-MNP is in the cytoplasm. Bottom: magnetic forces attract the GEF-MNP to the membrane, where it can catalyse GTPase activation. (c) COS7 cell co-expressing Rac1-GFP (green) and HaloTag–mCherry–TIAM1 (red). TIAM-MNPs appear as bright red spots and are highlighted by arrows. (d) Kymograph showing Rac1-GFP recruitment on top of a TIAM-MNP over time. Red bars indicate the presence of the magnetic tip. (e) Quantification of the Rac1-GFP recruitment at the TIAM-MNP surface. Reprinted by permission from Springer Nature, *Nature Nanotechnology*, “Subcellular control of Rac-GTPase signalling by magnetogenetic manipulation inside living cells”, Etoc, F.; Lisse, D.; Bellaiche, Y.; Piehler, J.; Coppey, M.; Dahan, M., copyright (2013).

An alternative to receptor clustering is to use MNPs as nanoscale platforms inside the cell to locally modulate intracellular functions in space or time.^120^ This strategy has been used to artificially modulate the remote-controlled arrest of mitochondrial dynamics^117^ or pathways implied in the nucleation of microtubules among others.^118,119^ For instance, Etoc *et al.* developed a magnetogenetic assay in which MNPs could specifically bind to tagged proteins inside cells, focusing their attention on two Rho-GTPases (Cdc42 and Rac1) involved in cell migration and polarization.^70^ To this end, 500 nm streptavidin-coated MNPs where modified with a biotinylated-HaloTag ligand that could covalently bind inside the cell (*in situ*) to overexpressed intracellular proteins fused to the HaloTag (Fig. 17a). Once attached to the proteins inside the living cells, MNPs are expected to behave as platforms, recruiting the signalling machinery and activating downstream pathways. Rac1 is a membrane-anchored protein that cycles between an “on” state when is bound to guanosine triphosphate (GTP) and an “off” state when is bound to guanosine diphosphate (GDP) (Fig. 17b). Rac1 is positively regulated by the guanine nucleotide exchange factor (GEF) TIAM 1 (T lymphoma invasion and metastasis 1). MNPs were injected into the cell and coupled *in situ* with a truncated domain of TIAM1^DHPH^ that lacked the ability to migrate to the cellular membrane; thus, they were localized in the cytoplasm and could not recruit Rac1. When using a magnetic tip to pull the MNPs to the membrane, Rac1 was reversibly recruited and activated, and actin polymerization was triggered around the MNPs (Fig. 17c–e). Later on, it was demonstrated that due to the viscoelastic properties of the cytoplasm, MNPs smaller than 50 nm could be also manipulated inside living cells at weak magnetic forces, in the femto-Newton range instead of in the pN range.^117^

Similarly, using smaller MNPs, this strategy has been exploited to capture in the cytoplasm SOS1, a GEF that can convert inactive RAS-GDP into active RAS-GTP.^121^ H-RAS is membrane-anchored protein belonging to the RAS family that mediates a signalling cascade related with cell survival, cell division and neurite outgrowth.^122^ In this case, 8 nm MNPs were coated with a silica shell and functionalised with an HaloTag ligand through click chemistry.^123^ After injecting the MNPs into the cytoplasm, they coupled intracellularly with HaloTag-fused SOS1. A magnetic tip was applied to guide the complex to the membrane where RAS1 was present, observing a reversible accumulation in the neurite tip. The main aim of activating the pathway triggered by RAS in the neurite is to provide an alternative method for direct axonal growth of neurons.

## 7. Magnetogenetics is not free of controversy

Despite its potential, the pathway on the development of magnetogenetics is not free of bumps. Some of the initial successful studies on magnetogenetics described, in 2015 and 2016, the magnetically sensitive actuators MAR,^109^ Magneto^13^ and TRPV1-ferritin^104^ constructs. After such works, different groups were able to use the same constructs successfully, reporting positive results using genetic techniques to excite neurons under the exposure to magnetic fields using the TRPV1 or TRPV4-ferritin constructs.^67,124,125^ Yet, magnetogenetics-based approaches claiming to have achieved neuronal activation, especially when relying on ferritin nanoparticles, faced some scepticism and criticism from the scientific community. In 2019 and 2020, three independent research groups^126–128^ questioned the earlier magnetogenetics results, as they were not able to replicate previous experiments using these constructs. In particular Kole *et al.*^126^ and Xu *et al.*^128^ performed experiments using the Magneto construct, while Wang *et al.*^127^ used Magneto, the TRPV1-ferritin complex and the MAR construct. These three works failed to obtain the initial results previously reported. These unsuccessful works have also been followed by a reply letter from the authors of one of the original works arguing about the differences between the initial work and the subsequent ones. In addition to such conflicting results, some theoretical approaches have deeply criticized the theories proposed to explain the obtained results,^129,130^ even arguing that such claims contradict basic laws of physics.^129^ Interestingly, other authors have proposed alternative hypothesis to explain the observed effects.^19,26^ In general, these alternative mechanisms are less studied than the mechanisms described in section 2.1. or even remain unexplored either experimentally or theoretically. These hypothetical mechanisms are:

**Diamagnetic force on the ion channel (Magnetic field: uniform DC)**. This proposed mechanism is based on the use of a DC field, able to orient the magnetic moment of the magnetic actuator, which at the same time would create a gradient on the ion channel/cell membrane generating a repulsive diamagnetic force.^26^

**Einstein-de Haas effect (Magnetic field: AC)**. In this effect, the reversal of the magnetic moment of a particle, by being exposed to an AC field, has to be accompanied by a change in the mechanical angular momentum of the particle, required for the conservation of the angular momentum. Therefore, the exposure of a magnetic actuator to an AC field would be also associated to a torque movement.^26^

**Magnetocaloric effect (Magnetic field: AC)**. The idea behind this mechanism is that particles would heat up during magnetisation and cool down during demagnetisation. This mechanism is based on the absorption of heat that occurs during demagnetisation when using very low frequency AC magnetic fields (<1 Hz). The demagnetisation process would cool the particle, allowing the activation of cold-activated ion channels.^125^

Another aspect that has been subject of controversy is the confinement of the heat generated by the MNPs on their close vicinity, as pointed out in several studies discussed in section 5 (see for instance Fig. 11b). Typically, such temperature measurements are based on the use of thermochromic dyes attached to the nanoparticle surface. In a recent study, Davis *et al.* questioned whether the results previously reported could be due to measurement artifacts and proposed a more reliable method based on the use of two different fluorescent dyes with well-separated emission maxima: one attached to the MNP and one dissolved in the surrounding medium.^66^ This approach allows for an independent, simultaneous measurement of both temperatures using an optical fibre. As opposed to previous claims, this method revealed practically no temperature difference between the surface of the nanoparticles and the surrounding media, independently of the size and composition of the MNPs, or the AMF conditions. Moreover, ferritin showed no measurable local or bulk heating under AMF exposure.

Taken together, these results suggest that a more in-depth evaluation of the mechanisms proposed to explain nanoscale magnetothermal activation would be advisable. In fact, the mechanism of action of some of these magnetogenetics constructs is not completely clear.

## 8. Conclusions and outlook

Magnetogenetics has raised high expectations from the research community. Magnetic fields can overcome the penetration depth limits of visible light, and do not require a permanent physical connection to the tissue/organ of interest, thus allowing minimally invasive procedures. The possibility of using them as a switch to activate the stimulus when required and in a truly tetherless mode, would result in several advantages with respect to other alternatives such as optogenetics or chemogenetics. In this regard, magnetothermal stimulation of temperature sensitive ion channels was thought to hold great promise for neurostimulation. Although the possibility to use this technology to activate neurons in mice has been smartly described, this approach is still rather limited by the response time, usually in the frame of several seconds.^58,131^ On the contrary, we believe that due to its faster responses, magnetomechanical stimulation is positioning itself as a real alternative to optogenetics.^53^ Despite this clear promise, the field of magnetogenetics is still in its infancy, and there are still many obstacles to be overcome. In our opinion, the following aspects require special consideration.

(i) Optimization of the magnetic actuator: one major source of controversy is associated to the magnetic actuators and the related working mechanisms, as discussed in section 7. The most heated debate generally refers to the use of ferritin, due to the small magnetic moment associated to the iron-containing ferritin core. However, such difficulties can be overcome if using MNPs. In fact, many of the magnetogenetics applications discussed in this review have clearly benefited from the recent advances in the field of Nanotechnology, mainly the synthesis and smart functionalisation of MNPs. By carefully selecting and tuning the synthetic procedure and adjusting its parameters, MNPs can be obtained practically with full control over their physicochemical properties (size, shape, composition, monodispersity, *etc*.).^132,133^ This in turn can translate into improved magnetic properties, which are critical for magnetogenetic actuation, as described in section 2.2 of this review. These advances in the preparation of magnetic actuators with tailored properties provide exciting opportunities for the field, as is for instance the case of using a mixture of MNPs with different sizes, shapes and compositions, each one susceptible for activation by a different combination of AMF amplitude and frequency. This concept, coined as “magnetothermal multiplexing”, could enable a selective remote control of different signalling processes, as recently demonstrated *in vitro* by Anikeeva and co-workers^49^ (Fig. 4, see also section 2.2). However, despite various robust synthetic methods being implemented at laboratory scale (with the most common being the co-precipitation and the thermal decomposition), large-scale production of reproducible MNPs for biomedical applications remains challenging.

(ii) Interaction with biological entities: besides having the required physical and chemical features needed for magnetomechanical and magnetothermal stimulation, the MNPs must be also biocompatible, colloidally stable and suitably functionalised to bind their specific cellular targets. In this regard, much effort has been put into developing robust MNP coating and functionalisation protocols tailored for specific bioapplications (for recent review articles, see for example ref. 134–136). Concerning cellular targets, in many magnetogenetic examples the receptors have been exogenously expressed in cells, which do not fully represent reality. It would be desirable to target receptors that are already present in the cells to avoid prior cell manipulation. In this case, since receptors are often expressed in various tissues, selectivity should be ensured to avoid off-target effects caused by the magnetic activation. Finally, and although we can find exciting examples of magnetogenetics performed *in vivo*, most of the work has been done using 2D *in vitro* models, which lack realistic complexity. In our opinion, the use of microfluidic devices and “organ-on-a-chip” approaches could be a way to investigate magnetogenetic stimulation under conditions that mimic in a more accurate fashion the *in vivo* scenario. This could allow an unprecedented wealth of information to be obtained in a short time, while reducing the costs and the number of animals for *in vivo* experimentation.

(iii) Standardization: Another limitation of magnetogenetics experiments is the fact that magnetic applicators are usually custom-made, as there is currently little commercial equipment available. Therefore, it is difficult to compare the results from different reports. Last, but not least, magnetogenetics would clearly benefit from more in-depth and systematic studies comparing the performance of diverse types of magnetic actuators to activate the same pathways.

Although the path is challenging, we are confident that addressing these issues could pave the way to achieve the high expectations initially raised by magnetogenetics.

## Conflicts of interest

There are no conflicts to declare.

## Supplementary Material

## References

[cit1] Jaalouk D. E., Lammerding J. (2009). Nat. Rev. Mol. Cell Biol..

[cit2] Repina N. A., Rosenbloom A., Mukherjee A., Schaffer D. V., Kane R. S. (2017). Annu. Rev. Chem. Biomol. Eng..

[cit3] Stanley S. A., Friedman J. M. (2019). Cold Spring Harb. Perspect. Med..

[cit4] Monzel C., Vicario C., Piehler J., Coppey M., Dahan M. (2017). Chem. Sci..

[cit5] Connell J. J., Patrick P. S., Yu Y., Lythgoe M. F., Kalber T. L. (2015). Regen. Med..

[cit6] Muhamed I., Chowdhury F., Maruthamuthu V. (2017). Bioengineering.

[cit7] Berret J. F. (2016). Nat. Commun..

[cit8] Seo D., Southard K. M., Kim J., Lee H. J., Farlow J., Lee J., Litt D. B., Haas T., Alivisatos A. P., Cheon J., Gartner Z. J., Jun Y. (2016). Cell.

[cit9] Kim J. W., Jeong H. K., Southard K. M., Jun Y. W., Cheon J. (2018). Acc. Chem. Res..

[cit10] Hughes S., McBain S., Dobson J., El Haj A. J. (2008). J. R. Soc., Interface.

[cit11] Mannix R. J., Kumar S., Cassiola F., Montoya-Zavala M., Feinstein E., Prentiss M., Ingber D. E. (2008). Nat. Nanotechnol..

[cit12] Cho M. H., Lee E. J., Son M., Lee J. H., Yoo D., Kim J. W., Park S. W., Shin J. S., Cheon J. (2012). Nat. Mater..

[cit13] Wheeler M. A., Smith C. J., Ottolini M., Barker B. S., Purohit A. M., Grippo R. M., Gaykema R. P., Spano A. J., Beenhakker M. P., Kucenas S., Patel M. K., Deppmann C. D., Güler A. D. (2016). Nat. Neurosci..

[cit14] Kang H., Wong D. S. H., Yan X., Jung H. J., Kim S., Lin S., Wei K., Li G., Dravid V. P., Bian L. (2017). ACS Nano.

[cit15] Rotherham M., Haj A. J. E. (2015). PLoS One.

[cit16] Tanase M., Biais N., Sheetz M. (2007). Methods Cell Biol..

[cit17] Lipfert J., Hao X., Dekker N. H. (2009). Biophys. J..

[cit18] Kilinc D., Lee G. U. (2014). Integr. Biol..

[cit19] Brier M. I., Mundell J. W., Yu X., Su L., Holmann A., Squeri J., Zhang B., Stanley S. A., Friedman J. M., Dordick J. S. (2020). Sci. Rep..

[cit20] Fleutot S., Nealon G. L., Pauly M., Pichon B. P., Leuvrey C., Drillon M., Gallani J. L., Guillon D., Donnio B., Begin-Colin S. (2013). Nanoscale.

[cit21] Coughlin M. F., Bielenberg D. R., Lenormand G., Marinkovic M., Waghorne C. G., Zetter B. R., Fredberg J. J. (2013). Clin. Exp. Metastasis.

[cit22] Carrey J., Hallali N. (2016). Phys. Rev. B.

[cit23] Hergt R., Dutz S., Müller R., Zeisberger M. (2006). J. Phys.: Condens. Matter.

[cit24] Beola L., Grazú V., Fernández-Afonso Y., Fratila R. M., De Las Heras M., De La Fuente J. M., Gutiérrez L., Asín L. (2021). ACS Appl. Mater. Interfaces.

[cit25] Mai T., Hilt J. Z. (2017). J. Nanopart. Res..

[cit26] Barbic M. (2019). eLife.

[cit27] Shin T.-H., Cheon J. (2017). Acc. Chem. Res..

[cit28] Bedanta S., Kleemann W. (2009). J. Phys. D: Appl. Phys..

[cit29] Leslie-Pelecky D. L., Rieke R. D. (1996). Chem. Mater..

[cit30] Huber D. (2005). Small.

[cit31] Yavuz C. T., Mayo J. T., Yu W. W., Prakash A., Falkner J. C., Yean S., Cong L., Shipley H. J., Kan A., Tomson M., Natelson D., Colvin V. L. (2006). Science.

[cit32] Butler R. F., Banerjee S. K. (1975). J. Geophys. Res..

[cit33] Song M., Kim J., Shin H., Kim Y., Jang H., Park Y., Kim S. J. (2020). Nanomaterials.

[cit34] Rotherham M., Nahar T., Goodman T., Telling N., Gates M., El Haj A. (2019). Adv. Biosyst..

[cit35] Rotherham M., Henstock J. R., Qutachi O., El Haj A. J. (2018). Nanomedicine.

[cit36] Hao L., Li L., Wang P., Wang Z., Shi X., Guo M., Zhang P. (2019). Nanoscale.

[cit37] Romero G., Christiansen M. G., Stocche Barbosa L., Garcia F., Anikeeva P. (2016). Adv. Funct. Mater..

[cit38] Hergt R., Dutz S. (2007). J. Magn. Magn. Mater..

[cit39] Stanley S. A., Gagner J. E., Damanpour S., Yoshida M., Dordick J. S., Friedman J. M. (2012). Science.

[cit40] Levy M., Quarta A., Espinosa A., Figuerola A., Wilhelm C., García-Hernández M., Genovese A., Falqui A., Alloyeau D., Buonsanti R., Cozzoli P. D., García M. A., Gazeau F., Pellegrino T. (2011). Chem. Mater..

[cit41] Guardia P., Riedinger A., Nitti S., Pugliese G., Marras S., Genovese A., Materia M. E., Lefevre C., Manna L., Pellegrino T. (2014). J. Mater. Chem. B.

[cit42] Jun Y. W., Huh Y. M., Choi J. S., Lee J. H., Song H. T., Kim S., Yoon S., Kim K. S., Shin J. S., Suh J. S., Cheon J. (2005). J. Am. Chem. Soc..

[cit43] Mathew D. S., Juang R.-S. (2007). Chem. Eng. J..

[cit44] Lee J.-H., Huh Y.-M., Jun Y., Seo J., Jang J., Song H.-T., Kim S., Cho E.-J., Yoon H.-G., Suh J.-S., Cheon J. (2007). Nat. Med..

[cit45] Jang J., Nah H., Lee J.-H., Moon S. H., Kim M. G., Cheon J. (2009). Angew. Chem., Int. Ed..

[cit46] Huang H., Delikanli S., Zeng H., Ferkey D. M., Pralle A. (2010). Nat. Nanotechnol..

[cit47] Lee J. H., Kim J. W., Levy M., Kao A., Noh S. H., Bozovic D., Cheon J. (2014). ACS Nano.

[cit48] Lee J.-H., Kim E. S., Cho M. H., Son M., Yeon S.-I., Shin J.-S., Cheon J. (2010). Angew. Chem..

[cit49] Moon J., Christiansen M. G., Rao S., Marcus C., Bono D. C., Rosenfeld D., Gregurec D., Varnavides G., Chiang P. H., Park S., Anikeeva P. (2020). Adv. Funct. Mater..

[cit50] Munshi R., Qadri S. M., Pralle A. (2018). Front. Neurosci..

[cit51] Guardia P., Di Corato R., Lartigue L., Wilhelm C., Espinosa A., Garcia-Hernandez M., Gazeau F., Manna L., Pellegrino T. (2012). ACS Nano.

[cit52] Gregurec D., Senko A. W., Chuvilin A., Reddy P. D., Sankararaman A., Rosenfeld D., Chiang P., Garcia F., Tafel I., Varnavides G., Ciocan E., Anikeeva P. (2020). ACS Nano.

[cit53] uk Lee J., Shin W., Lim Y., Kim J., Kim W. R., Kim H., Lee J. H., Cheon J. (2021). Nat. Mater..

[cit54] Kim J. W., Lee J. H., Ma J. H., Chung E., Choi H., Bok J., Cheon J. (2016). Nano Lett..

[cit55] Tay A., Di Carlo D. (2017). Nano Lett..

[cit56] Kunze A., Tseng P., Godzich C., Murray C., Caputo A., Schweizer F. E., Di Carlo D. (2015). ACS Nano.

[cit57] Lee J.-H., Jang J., Choi J., Moon S. H., Noh S., Kim J., Kim J.-G., Kim I.-S., Park K. I., Cheon J. (2011). Nat. Nanotechnol..

[cit58] Munshi R., Qadri S. M., Zhang Q., Rubio I. C., del Pino P., Pralle A. (2017). eLife.

[cit59] Gutiérrez L., Barrón V., Andrés-Vergés M., Serna C. J., Veintemillas-Verdaguer S., Morales M. P., Lázaro F. J. (2016). J. Geophys. Res. Solid Earth.

[cit60] Manceau A. (2011). Am. Mineral..

[cit61] Michel F. M., Barrón V., Torrent J., Morales M. P., Serna C. J., Boily J. F., Liu Q., Ambrosini A., Cismasu A. C., Brown G. E. (2010). Proc. Natl. Acad. Sci. U. S. A..

[cit62] López-Castro J. D., Delgado J. J., Perez-Omil J. A., Gálvez N., Cuesta R., Watt R. K., Domínguez-Vera J. M. (2012). Dalton Trans..

[cit63] Gutiérrez L., Lázaro F. J., Abadía A. R., Romero M. S., Quintana C., Morales M. P., Patiño C., Arranz R. (2006). J. Inorg. Biochem..

[cit64] Liße D., Monzel C., Vicario C., Manzi J., Maurin I., Coppey M., Piehler J., Dahan M. (2017). Adv. Mater..

[cit65] Ducasse R., Wang W. A., Navarro M. G. J., Debons N., Colin A., Gautier J., Guigner J. M., Guyot F., Gueroui Z. (2017). Sci. Rep..

[cit66] Davis H. C., Kang S., Lee J. H., Shin T. H., Putterman H., Cheon J., Shapiro M. G. (2020). Biophys. J..

[cit67] Hutson M. R., Keyte A. L., Hernández-Morales M., Gibbs E., Kupchinsky Z. A., Argyridis I., Erwin K. N., Pegram K., Kneifel M., Rosenberg P. B., Matak P., Xie L., Grandl J., Davis E. E., Katsanis N., Liu C., Benner E. J. (2017). Sci. Signal..

[cit68] Vogel V. (2006). Annu. Rev. Biophys. Biomol. Struct..

[cit69] Wang N., Tytell J. D., Ingber D. E. (2009). Nat. Rev. Mol. Cell Biol..

[cit70] Etoc F., Lisse D., Bellaiche Y., Piehler J., Coppey M., Dahan M. (2013). Nat. Nanotechnol..

[cit71] Lovendahl K. N., Blacklow S. C., Gordon W. R. (2018). Adv. Exp. Med. Biol..

[cit72] Moore S. W., Roca-Cusachs P., Sheetz M. P. (2010). Dev. Cell.

[cit73] Askari J. A., Buckley P. A., Mould A. P., Humphries M. J. (2009). J. Cell Sci..

[cit74] Barczyk M., Carracedo S., Gullberg D. (2010). Cell Tissue Res..

[cit75] Geiger B., Bershadsky A., Pankov R., Yamada K. M. (2001). Nat. Rev. Mol. Cell Biol..

[cit76] LaFlamme S. E., Mathew-Steiner S., Singh N., Colello-Borges D., Nieves B. (2018). Cell. Mol. Life Sci..

[cit77] Atherton P., Stutchbury B., Jethwa D., Ballestrem C. (2016). Exp. Cell Res..

[cit78] Chakraborty S., Banerjee S., Raina M., Haldar S. (2019). Biochemistry.

[cit79] Pawar A., Balasubramanian N. (2017). J. Indian Inst. Sci..

[cit80] Cartmell S. H., Dobson J., Verschueren S. B., El Haj A. J. (2002). IEEE Trans. Nanobioscience.

[cit81] El Haj A. J., Glossop J. R., Sura H. S., Lees M. R., Hu B., Wolbank S., van Griensven M., Redl H., Dobson J. (2015). J. Tissue Eng. Regener. Med..

[cit82] Kanczler J. M., Sura H. S., Magnay J., Green D., Oreffo R. O. C., Dobson J. P., El Haj A. J. (2010). Tissue Eng., Part A.

[cit83] Maître J. L., Heisenberg C. P. (2013). Curr. Biol..

[cit84] Leckband D. E., de Rooij J. (2014). Annu. Rev. Cell Dev. Biol..

[cit85] Barcelona-Estaje E., Dalby M. J., Cantini M., Salmeron-Sanchez M. (2021). Adv. Healthcare Mater..

[cit86] Kwak M., Gu W., Jeong H., Lee H., Lee J. U., An M., Kim Y. H., Lee J. H., Cheon J., Jun Y. W. (2019). Nano Lett..

[cit87] Andersson E. R., Sandberg R., Lendahl U. (2011). Development.

[cit88] Nusse R. (2008). Cell Res..

[cit89] Clevers H. (2006). Cell.

[cit90] Logan C. Y., Nusse R. (2004). Annu. Rev. Cell Dev. Biol..

[cit91] Milat F., Ng K. W. (2009). Mol. Cell. Endocrinol..

[cit92] Jin P., Jan L. Y., Jan Y. N. (2020). Annu. Rev. Neurosci..

[cit93] Shen Y., Cheng Y., Uyeda T. Q. P., Plaza G. R. (2017). Ann. Biomed. Eng..

[cit94] Cox C. D., Bavi N., Martinac B. (2019). Cell Rep..

[cit95] Hughes S., Magnay J., Foreman M., Publicover S. J., Dobson J. P., El Haj A. J. (2006). J. Cell. Physiol..

[cit96] Henstock J. R., Rotherham M., Rashidi H., Shakesheff K. M., El Haj A. J. (2014). Stem Cells Transl. Med..

[cit97] Henstock J. R., Rotherham M., El Haj A. J. (2018). J. Tissue Eng..

[cit98] Wu J., Goyal R., Grandl J. (2016). Nat. Commun..

[cit99] Clapham D. E. (2003). Nature.

[cit100] Lamas J. A., Rueda-Ruzafa L., Herrera-Pérez S. (2019). Int. J. Mol. Sci..

[cit101] Samanta A., Hughes T. E. T., Moiseenkova-Bell V. Y. (2018). Sub-cellular biochemistry.

[cit102] Nimpf S., Keays D. A. (2017). EMBO J..

[cit103] Güler A. D., Lee H., Iida T., Shimizu I., Tominaga M., Caterina M. (2002). J. Neurosci..

[cit104] Stanley S. A., Kelly L., Latcha K. N., Schmidt S. F., Yu X., Nectow A. R., Sauer J., Dyke J. P., Dordick J. S., Friedman J. M. (2016). Nature.

[cit105] Wu S., Li H., Wang D., Zhao L., Qiao X., Zhang X., Liu W., Wang C., Zhou J. (2021). Nano Today.

[cit106] Tay A., Kunze A., Murray C., Di Carlo D. (2016). ACS Nano.

[cit107] Hughes S., El Haj A. J., Dobson J. (2005). Med. Eng. Phys..

[cit108] Dobson J., Cartmell S. H., Keramane A., El Haj A. J. (2006). IEEE Trans. Nanobioscience.

[cit109] Long X., Ye J., Zhao D., Zhang S. J. (2015). Sci. Bull..

[cit110] Stanley S. A., Sauer J., Kane R. S., Dordick J. S., Friedman J. M. (2015). Nat. Med..

[cit111] Chen R., Romero G., Christiansen M. G., Mohr A., Anikeeva P. (2015). Science.

[cit112] Hunt S. P., Pini A., Evan G. (1987). Nature.

[cit113] Cho M. H., Kim S., Lee J. H., Shin T. H., Yoo D., Cheon J. (2016). Nano Lett..

[cit114] Bharde A. A., Palankar R., Fritsch C., Klaver A., Kanger J. S., Jovin T. M., Arndt-Jovin D. J. (2013). PLoS One.

[cit115] Perica K., Tu A., Richter A., Bieler J. G., Edidin M., Schneck J. P. (2014). ACS Nano.

[cit116] Kosmides A. K., Necochea K., Hickey J. W., Schneck J. P. (2018). Nano Lett..

[cit117] Etoc F., Vicario C., Lisse D., Siaugue J. M., Piehler J., Coppey M., Dahan M. (2015). Nano Lett..

[cit118] Bonnemay L., Hostachy S., Hoffmann C., Gautier J., Gueroui Z. (2013). Nano Lett..

[cit119] Hoffmann C., Mazari E., Lallet S., Le Borgne R., Marchi V., Gosse C., Gueroui Z. (2013). Nat. Nanotechnol..

[cit120] Bonnemay L., Hoffmann C., Gueroui Z. (2015). Wiley Interdiscip. Rev. Nanomed. Nanobiotechnol..

[cit121] Raudzus F., Schöneborn H., Neumann S., Secret E., Michel A., Fresnais J., Brylski O., Ménager C., Siaugue J. M., Heumann R. (2020). Sci. Rep..

[cit122] Talebian A., Robinson-Brookes K., MacDonald J. I. S., Meakin S. O. (2013). J. Mol. Neurosci..

[cit123] Schöneborn H., Raudzus F., Secret E., Otten N., Michel A., Fresnais J., Ménager C., Siaugue J.-M., Zaehres H., Dietzel I. D., Heumann R. (2019). J. Funct. Biomater..

[cit124] Mosabbir A. A., Truong K. (2018). ACS Synth. Biol..

[cit125] Duret G., Polali S., Anderson E. D., Bell A. M., Tzouanas C. N., Avants B. W., Robinson J. T. (2019). Biophys. J..

[cit126] Kole K., Zhang Y., Jansen E. J. R., Brouns T., Bijlsma A., Calcini N., Yan X., Lantyer A. da S., Celikel T. (2020). Nat. Neurosci..

[cit127] Wang G., Zhang P., Mendu S. K., Wang Y., Zhang Y., Kang X., Desai B. N., Zhu J. J. (2020). Nat. Neurosci..

[cit128] Xu F. X., Zhou L., Wang X. T., Jia F., Ma K. Y., Wang N., Lin L., Xu F. Q., Shen Y. (2020). Nat. Neurosci..

[cit129] Meister M. (2016). eLife.

[cit130] Anikeeva P., Jasanoff A. (2016). eLife.

[cit131] Mushi R., Pralle A. (2021). Nat. Mater..

[cit132] Roca A. G., Gutiérrez L., Gavilán H., Fortes Brollo M. E., Veintemillas-Verdaguer S., Morales M. P. (2019). Adv. Drug Delivery Rev..

[cit133] Senthilkumar N., Sharma P. K., Sood N., Bhalla N. (2021). Coord. Chem. Rev..

[cit134] Bohara R. A., Thorat N. D., Pawar S. H. (2016). RSC Adv..

[cit135] Cardoso V. F., Francesko A., Ribeiro C., Bañobre-López M., Martins P., Lanceros-Mendez S. (2018). Adv. Healthcare Mater..

[cit136] Dadfar S. M., Roemhild K., Drude N. I., von Stillfried S., Knüchel R., Kiessling F., Lammers T. (2019). Adv. Drug Delivery Rev..

[cit137] Hu B., El Haj A. J., Dobson J. (2013). Int. J. Mol. Sci..

